# RECQ5 mediates pre-rRNA processing in nucleolus

**DOI:** 10.1093/nar/gkaf766

**Published:** 2025-08-18

**Authors:** Yidi Ma, Xiaochun Yu

**Affiliations:** College of Life Sciences, Zhejiang University, Hangzhou, Zhejiang, 310058, China; Westlake Laboratory of Life Sciences and Biomedicine, Hangzhou, Zhejiang, 310024, China; School of Life Sciences, Westlake University, Hangzhou, Zhejiang, 310030, China; Institute of Basic Medical Sciences, Westlake Institute for Advanced Study, Hangzhou, Zhejiang Province, 310024, China; Westlake Laboratory of Life Sciences and Biomedicine, Hangzhou, Zhejiang, 310024, China; School of Life Sciences, Westlake University, Hangzhou, Zhejiang, 310030, China; Institute of Basic Medical Sciences, Westlake Institute for Advanced Study, Hangzhou, Zhejiang Province, 310024, China

## Abstract

RECQ5 is a member of the RECQ helicase family that maintains genomic stability. However, the molecular mechanism of RECQ5 in this biological process remains elusive. Here, we show that RECQ5 localizes in the dense fibrillar component of nucleolus and associates with several pre-rRNA processing factors. It recognizes pre-rRNA and is able to unwind double-stranded RNA *in vitro*. Loss of RECQ5 induces the accumulation of 47S, 30SL5′, and 30S pre-rRNA, and the reduction of 21S pre-rRNA, suggesting that it regulates the processing of pre-rRNA. Cancer-associated mutations of RECQ5 abolish its nucleolar localization as well as its helicase activities. Moreover, lacking RECQ5 leads to the unprocessed pre-rRNA hybridizing with rDNA, triggering the R-loop formation and ATR activation. Since both RECQ5 and ATR protect genomic stability in nucleolus, suppression of RECQ5 sensitizes tumor cells to the ATR inhibitor treatment. Collectively, this study reveals that RECQ5 plays a crucial role in pre-rRNA processing and ribosomal DNA (rDNA) stability.

## Graphical Abstract

**Graphical Abstract:**
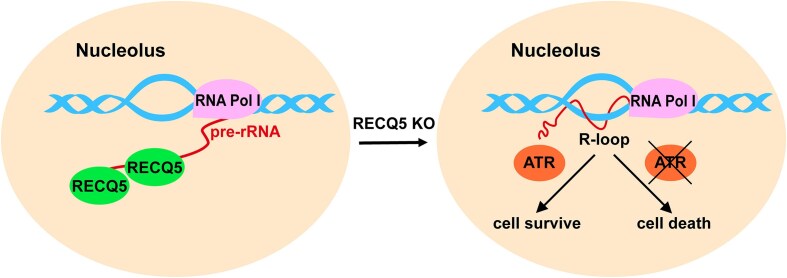

## Introduction

RECQ helicase family is one of the most conserved groups of 3′–5′ helicases and is named after the prototype *Escherichia coli* RecQ [1]. There are five members in human cells, namely RECQ1, BLM/RECQ2, WRN/RECQ3, RECQ4, and RECQ5. Most of these gene products share a conserved helicase fold containing the core ATPase domain and the RECQ C-terminal (RQC) domain [2]. The RQC domain recognizes nucleic acid substrates, while the core ATPase domain consumes ATP for substrate unwinding [3–5]. Thus, both the ATPase domain and the RQC domain are essential for helicase activity.

The helicase activities of RECQ family enzymes play important roles in maintaining genomic stability [5]. For example, BLM is a key subunit in a Holliday junction resolvase that unwinds and digests Holliday junctions during homologous recombination (HR) repair [6, 7]. Thus, the mutations of BLM are associated with Bloom’s syndrome, a genetic disorder with genomic instability and tumorigenesis [8]. Likewise, mutations of WRN and RECQ4 are associated with Werner’s syndrome and Rothmund–Thomson syndrome, respectively [9]. Similar to other family members, it has been shown that RECQ5 participates in DNA double-strand break (DSB) repair by directly interacting with RAD51 during HR, and functions in genome stability and transcriptional regulation via association with RNA polymerase I (Pol I) or RNA polymerase II [10–12]. Thus, RECQ5 may have multiple functions in different biological processes to maintain genomic stability.

Moreover, germline mutations of RECQ5 have been identified in familial breast cancer patients [13]. These mutations are either missense mutations in the helicase domain or insertion/deletion mutations that abolish the helicase domain of RECQ5. Sporadic mutations of RECQ5 have also been found in various types of cancer [14–16]. Thus, RECQ5 is an important tumor suppressor.

RECQ helicases are evolutionarily conserved from prokaryotes to eukaryotes [17]. The ortholog of RECQ helicases in *Saccharomyces cerevisiae* is Sgs1, which also maintains genomic stability [18, 19]. This biological function is particularly manifested at ribosomal DNA (rDNA) loci, because deletion of Sgs1 induces extrachromosomal circular DNA formation that depletes rDNA copies and accelerates cellular senescence [20]. However, as an ortholog, it is unclear whether RECQ5 regulates genomic stability at rDNA loci in human cells. Here, we found that RECQ5 existed in nucleolus and regulated pre-rRNA processing. Loss of RECQ5 causes genome instability at rDNA loci by inducing the accumulation of RNA/DNA hybrids.

## Materials and methods

### Cell culture

All the cells were cultured according to the directions from ATCC. HeLa and MCF-7 cells were cultured in the high-glucose Dulbecco’s modified Eagle’s medium (DMEM) supplemented with 10% Fetal Bovine Serum (FBS). MDA-MB-231 cells were cultured in the high-glucose DMEM medium supplemented with 15% FBS. U2OS cells were cultured in McCoy’s 5A medium supplemented with 10% FBS.

### Antibodies and reagents

Detailed information of antibodies used in this study: anti-POLR1A (Proteintech, Rabbit, 20595-1-AP), anti-DKC1 (Santa Cruz, Mouse, sc-373956), anti-RECQ5 (Novus, Rabbit, NBP1-83438; CST, Mouse, 5847S; Abcam, Rabbit, ab224135), anti-Nucleolin (Invitrogen, Mouse, ZN004), anti-FBL (CST, Rabbit, 2639S; Abcam, Rabbit, ab5821), anti-NOP56 (AiFang biological, Rabbit, AF07227; Invitrogen, Mouse, MA5-24641), anti-NPM1 (Invitrogen, Mouse, MA5-12508), IgG (Rabbit, CST, 2729), IgG (Mouse, CST, 3420), anti-GFP (Proteintech, Mouse, 66002-1-Ig), anti-γH2AX (Home-made, Mouse), anti-S9.6 (Kerafast, Mouse, ENH001; Merck, Mouse, MABE1095), anti-GAPDH (HUABIO, Rabbit, ET1601-4); anti-ATR (Bethyl, Rabbit, A300-137A), anti-CHK1 (Novus, Rabbit, NB100-464), anti-pATR (T1989) (GeneTex, Rabbit, GTX128145), anti-pCHK1 (Ser345) (CST, Rabbit, 2348), anti-TOPBP1 (Santa Cruz, Mouse, sc-271043), anti-APE1 (Santa Cruz, Mouse, sc-17774), VE-821(MCE, HY-14731), RECQ5-IN-1(RECQ5 inhibitor, MCE, HY-145685), BMH-21 (Selleck, S7718), ATP-γ-S (MCE, HY-10866), BLM recombinant protein (690–1291 aa, ICE Bioscience Inc., E2202T-H19H), DDX3X recombinant protein (Nanjing Detai Bioengineering Co., Ltd, DTD0126).

### RECQ5 expression and knockout

To express EGFP–RECQ5, the full-length RECQ5 complementary DNA (cDNA) was inserted into the pEGFP-N1 vector. The mutants of RECQ5 were generated using Mut Express II Fast Mutagenesis Kit V2 (Vazyme). For ectopic expression, the plasmids were added to cells at ∼40% confluence via Lipofectamine 2000 Transfection Reagent (Invitrogen) according to the manufacturer’s protocol and cultured for 48 h. The cDNA encoding human RNaseH1 was integrated into the pEGFP-N1 vector for the ectopic expression in mammalian cells. RECQ5 was knocked out in HeLa and U2OS cells using CRISPR/Cas9 technology with guide RNA (gRNA) targeting the *RECQ5* gene (5′- TGAGCGGCGAGTCCGGAGT-3′). The sequences of oligonucleotide primers for small interfering RNA (siRNA) are listed in Supplementary Table S4.

### Protein expression and purification

To generate recombinant RECQ5 and its mutants, previously described pTXB1-based vectors were used [21]. Plasmids were transformed into *E. coli* BL21-CodonPlus (DE3)-RIPL strain and protein expression was induced with 0.4 mM Isopropyl β-D-thiogalactoside (IPTG), followed by the incubation at 16°C. Cells were pelleted and stored at −80°C. Cell pellets were resuspended in 50 ml of cell lysis buffer [20 mM Tris–HCl at pH 8.0, 500 mM NaCl, 1 mM ethylenediaminetetraacetic acid (EDTA), 0.1% (v/v) Triton X-100] supplemented with 0.2 mM Phenylmethylsulfonyl fluoride (PMSF) and incubated for 30 min at 4°C. Then, the cell lysates were sonicated for 10 min at 10 s on/20 s off at 4°C. After sonication, the cell lysates were clarified by centrifugation at 10 000 rpm for 1 h at 4°C. The supernatant was incubated for 2 h with 2 ml of chitin beads (New England Biolabs) preequilibrated in cell lysis buffer. Beads were washed with lysis buffer for five times. To induce intein cleavage, cleavage buffer [20 mM Tris–HCl at pH 8.0, 150 mM NaCl, 1 mM EDTA, 50 mM dithiothreitol (DTT)] was added to the beads and the beads were incubated on a rotary shaker O/N at 4°C overnight. Then, proteins were eluted with elution buffer [25 mM Tris–HCl at pH 8.5, 10% (v/v) glycerol, 0.5 mM EDTA, 150 mM KCl, 0.01% (v/v) NP40, and 1 mM DTT] at 4°C. The buffer containing RECQ5 was concentrated on a centrifugal concentrator (Thermo Scientific™ Pierce™ PES, 10K MWCO), aliquoted, and stored at −80°C.

### Stellaris RNA FISH

Stellaris RNA FISH (fluorescence *in situ* hybridization) and immunofluorescence (IF) were performed according to the manufacturer’s protocol [22]. Briefly, cells were grown on glass coverslips. After washing with phosphate buffered saline (PBS), the cells were fixed with 4% paraformaldehyde for 15 min and permeabilized with 0.5% Triton X-100 in PBS for 5 min at room temperature. After washing with PBS, cells were rehydrated with wash buffer A at room temperature for 5 min. Then, the cells were incubated with a hybridization buffer containing RNA FISH pre-rRNA probe and set in a humidified chamber overnight at 37°C. Then, the cells were washed once with wash buffer A at room temperature for 30 min and subjected to IF staining. After washing with PBS, the cells were incubated with primary antibodies and diluted in 3% bovine serum albumin (BSA). The cells were then washed with PBST (PBS with 0.2% Tween 20) and incubated with secondary antibodies. The coverslips were mounted in DAPI Fluoromount-G Antifade Mounting Medium (Yeasen). RNA FISH probes were with Cy3 on the 3′ ends as previously described [23].The Cy3-labeled probes were as follows:

AACCTCTCCGACGACAGG

AGAGGACAGCGTGTCAGC

CCGCGCGCATCCGGAGGC

GTCACCGGTAGGCCAGAG

GGAGCGCGGCCGGCTAGC

CCCGGCAGGCGGCTCAAG

GAGAGAACAGCAGGCCCG

AGTCGGGACGCTCGGACG

GGACCCGGGCCGGCACCG

CCCGGGTGGGTCAGAGAC

TCGCCCCCTTCCCCGCCG

CGCACGGGGGCACGGTGG

CGGGCGCCCGCAGCGGAG

CGGGGTGGGGTTGTCGCG

ACACGCACGGCACGGAGC

CGCGGAGACGAGAACCCC

GAAGGGGCGGCGGACAAC

GGCCAACCCCCCACTCCG

CCAGCGAGCCGATCGGCT

AGCGGAGGCCGGCCGGCC

### Structured illumination microscopy procedure

Structured illumination microscopy (SIM) images were acquired on a Multi-SIM (multimodality structured illumination microscopy) imaging system (NanoInsights-Tech Co., Ltd.) equipped with a 100× 1.49 NA oil objective (Nikon CFI SR HP Apo) and a Photometrics Kinetix camera. The SIM images were acquired using single-slice mode with a 50-mW laser power and a 30-ms exposure time. Images were then reconstructed using the SIM Imaging Analysis software (NanoInsights-Tech). For colocalization analysis, processing and quantitative measurements of fluorescence intensities were performed with ImageJ.

### Immunoprecipitation, mass spectrometry, and western blotting

Cell lysates were prepared by incubating cells in NETN 300 buffer (50 mM Tris–HCl at pH 8.0, 300 mM NaCl, 0.2% NP-40, 2 mM EDTA) in the presence of Protease Inhibitor Cocktails (Roche) and 25 U/ml DNase I (Beyotime) for 15 min at 4°C, followed by centrifugation at 12 000 × *g* for 15 min at 4°C and diluted with the same volume of NETN 0 buffer (50 mM Tris–HCl at pH 8.0, 0.2% NP-40, 2 mM EDTA). Then, the cell lysates were incubated with control or RECQ5 antibody (CST, Mouse, 5847S) at 4°C overnight with constant rotation, and the incubation was continued with Protein A/G Beads (Smart-Lifesciences) for 2 h. Then, the beads were washed three times with NETN 300 buffer and three times with NETN 150 buffer. Subsequently, the immunoprecipitated protein samples were run on an SDS–PAGE (sodium dodecyl sulfate–polyacrylamide gel electrophoresis) gel and the in-gel enzymatic digestion procedure was performed. The resulting peptides were subsequently analyzed using Thermo Exploris 480 for data acquisition. Data files from the Liquid Chromatography-Tandem Mass Spectrometry (LC–MS/MS ) analysis were processed using Proteome Discoverer (version 2.5) to identify proteins. Protein identifications required at least two unique peptides and excluded peptides and proteins with a false discovery rate (FDR) exceeding 1%. The immunoprecipitated proteins were analyzed by label-free quantification mass spectrometry. For western blotting assays, the samples were analyzed with the indicated antibodies.

### RNA isolation and northern blotting

Total RNAs from an equal number of nontreated or treated cells were extracted with Trizol Reagent (Invitrogen) according to the manufacturer’s protocol. Precipitated RNA was subjected to northern blotting to examine intermediates of pre-rRNAs. Briefly, RNA was separated on 1.5% denatured agarose gel in MOPS buffer, and then transferred to positively charged Amersham™ Hybond™-N+ Membranes (GE Healthcare) in 20× SSC buffer overnight. Then, the membranes were cross-linked with 0.12 J/cm^2^ 254 nm UV and dried at 80°C for 30 min. After pre-hybridization with Ambion^®^ ULTRAhyb^®^-Oligo buffer for 30 min, the membranes were hybridized with different biotin-labeled probes for at least 3 h. Finally, the signal was detected with the Chemiluminescent Nucleic Acid Detection Module Kit (Thermo Fisher Scientific). The biotin-labeled probes were as follows:

5′ETS-Biotin, CGGAGGCCCAACCTCTCCGACGACAGGTCGCCAGAGGACA

GCGTGTCAGC;

ITS1-Biotin, CCTCGCCCTCCGGGCTCCGTTAATGATC;

ITS2-Biotin, CTGCGAGGGAACCCCCAGCCGCGCA;

28S-Biotin, AACGATCAGAGTAGTGGTATTTCACC;

GAPDH-Biotin, AGGCGCCCAATACGACCAAATCCGTTGACT;

AGCGTCAAAGGTGGAGGAGTGGGTGTCGCT.

### Detection of nascent pre-rRNA and RT-qPCR

HeLa cells were pretreated with 20 μM 5-fluorouracil (5-FU, Sigma) for 3 h to arrest pre-rRNA processing and then pulse-chased with 100 μM 4-thiouridine (4sU, Sigma) for the final 30 min to monitor nascent pre-rRNA [24]. Total RNA was extracted and biotinylated in biotinylation buffer (10 mM Tris, pH 7.5, 1 mM EDTA) with 0.2 mg/ml EZ-Link Biotin-HPDP (Pierce) at room temperature for 1.5 h. Biotinylated RNA was incubated with streptavidin magnetic beads (Invitrogen) at room temperature for 30 min. The beads were subsequently washed four times with a 65°C washing buffer (100 mM Tris, pH 7.5, 10 mM EDTA, 1 M NaCl, 0.1% Tween 20) and four times with a room temperature washing buffer. Nascent RNA was eluted twice with 0.1 M DTT and was precipitated with 40 μl of 4 M LiCl, 2 μl glycogen, and 600 μl of ice-cold ethanol. Then, Reverse Transcription-Quantitative PCR (RT-qPCR) was performed to quantify the nascent pre-rRNA. Specifically, the cDNA was synthesized using ABScript III RT Master Mix for quantitative PCR (qPCR) with genomic DNA (gDNA) Remover (ABclonal). qPCR was performed with Hieff^®^ SYBR Green Master Mix (Yeasen) according to instructions. qPCR reactions were run on SIA-PCR007 (Biorad CFX Connect) and cycle program of 95°C for 2 min followed by 40 cycles with 95°C for 10 s and 60°C for 30 s. Pre-rRNA signals were normalized to actin signals. 2 ^(−ΔΔCt)^ was used to calculate fold changes.

The primers were as follows:

pre-rRNA-F: TGTCAGGCGTTCTCGTCTC

pre-rRNA-R: AGCACGACGTCACCACATC

actin-F: GGACTTCGAGCAAGAGATGG

actin-R: AGCACTGTGTTGGCGTACAG

### *In vitro* helicase assay

Helicase assay was performed as described previously [25]. Briefly, 50 nM of 5′ end Cy5-labeled substrates was incubated for 30 min with the purified RECQ5 in unwinding reaction buffer (25 mM MOPS at pH 7.0, 50 mM NaCl, 2 mM β-mercaptoethanol, 100 μg/ml BSA, 0.1 mM EDTA), followed by the addition of ATP (10 mM), MgCl_2_ (20 mM), and Trapping oligo (300 nM). After incubation of the mixture for 30 min at 37°C, reactions were terminated by the addition of stop buffer and analyzed by electrophoresis on 20% nondenaturing polyacrylamide gels. Products were visualized by Amersham Typhoon 5 and quantified using ImageJ software. Sequences of the substrates were listed in Supplementary Table S3.

### ATPase assay

First, purified RECQ5 diluted with reaction buffer (40 mM Tris–HCl at pH 7.5, 20 mM MgCl_2_, 100 μg/ml BSA) was dispensed to a 384-well white microplate, followed by the addition of RNA or DNA (100 nM) and ATP (1 mM) to initiate the reaction and incubated at room temperature. At the different time points, ATP hydrolysis was measured using ADP-Glo Kit (Promega) by adding 5 μl ADP-Glo reagent for 40 min, followed by 10 μl Kinase Detection Reagent for 1 h. Luminescence was measured with the Varioskan LUX Microplate Reader (Thermo Fisher Scientific).

### Immunofluorescence

Cells were seeded on glass slides, washed once with PBS, and then fixed with 4% paraformaldehyde for 15 min, and permeabilized with 0.5% Triton X-100 in PBS for 7 min. Samples were blocked in 3% BSA for 1 h and stained with primary antibodies diluted with 3% BSA overnight at 4°C. PBS was subsequently used to wash the slides three times. The slides were then incubated with secondary antibodies for 1 h at room temperature. The slides were mounted in a DAPI Fluoromount-G antifade mounting medium (Yeasen). For 5′ EU incorporation, cells were treated with 1 mM 5′ EU for 30 min. Then, the cells were fixed with 3% paraformaldehyde for 5 min and permeabilized with 0.5% Triton X-100 in PBS for 5 min. Samples were stained with click reaction buffer (800 μl of 100 mM Tris–HCl at pH 8.5, 10 μl of 5 mM azide-fluor 488 solution, 10 μl of 100 mM CuSO_4_ solution, 200 μl of 0.5 M ascorbic acid in water) for 30 min at room temperature. The intensity of the 5′ EU signal was quantified using ImageJ software. For S9.6 IF, cells were fixed with methanol for 20 min at −20°C. Then, the fixed cells were stained with S9.6 antibody diluted with blocking buffer (1% BSA and 0.3% Triton X-100 in PBS) overnight at 4°C, followed by secondary antibodies for 1 h at room temperature. The intensity of the S9.6 signal was quantified using ImageJ software.

### DNA–RNA hybrid Immunoprecipitation

DNA–RNA hybrid immunoprecipitation (DRIP) assays were performed as described previously [26]. Cells were fixed in 0.75% formaldehyde for 10 min at room temperature and 0.125 M glycine was used to quench cross-linking for 5 min. Cells were washed once with cold PBS and lysed with hypotonic buffer (20 mM Tris–HCl at pH 7.4, 10 mM NaCl, 3 mM MgCl_2,_ 0.1% NP-40) for 15 min on ice. The pellets were resuspended in R-loop digestion buffer (10 mM Tris–HCl at pH 8.0, 100 mM NaCl, 25 mM EDTA, 0.5% SDS, 0.2 mg/ml Proteinase K) and incubated overnight in a 65°C water bath. Then, 25:24:1 phenol:chloroform:isoamyl alcohol was added to extract DNA. The purified DNA was dissolved into the FA Lysis Buffer (20 mM Tris–HCl at pH 7.5, 150 mM NaCl, 1 mM Na_2_EDTA, 1 mM EGTA, 1% Triton, 2.5 mM sodium pyrophosphate, 1 mM β-glycerophosphate) before sonication. Sonication was completed to form an average fragment size of ∼500 bp of DNA (Bioruptor Pico, Diagenode), followed by incubation at 37°C overnight in the absence or presence of RNaseH1. Then, the DNA was immunoprecipitated overnight at 4°C with 10 μg of S9.6 antibody or anti-mouse IgG. Immunoprecipitated samples were bound to protein A/G Sepharose for 2 h and washed three times with washing buffer and washed once with ice-cold PBS buffer. After elution, 5 μl of proteinase K was added to each tube and allowed overnight incubation in a 65°C water bath. DNA was purified and quantified by qPCR using SYBR Green Master Mix on CFX Connect Real-Time PCR Detection System (Biorad). The sequence of primers (5′-3′) were as follows:

P1-F CCCGGGGGAGGTATATCTTT

P1-R CCAACCTCTCCAGCGACA

P2-F GGCGGTTTGAGTGAGACGAGA

P2-R ACGTGCGCTCACCGAGAGCAG

P3-F CGACGACCCATTCGAACGTCT

P3-R CTCTCCGGAATCGAACCCTGA

P4-F AGTCGGGTTGCTTGGGAATGC

P4-R CCCTTACGGTACTTGTTGACT

P5-F ACCTGGCGCTAAACCATTCGT

P5-R GGACAAACCCTTGTGTCGAGG

P6-F GTTGACGTACAGGGTGGACTG

P6-R GGAAGTTGTCTTCACGCCTGA

### Chromatin immunoprecipitation

Cells were fixed with 0.75% formaldehyde for 10 min at room temperature, quenched with 0.125 M glycine, and lysed in ChIP (chromatin immunoprecipitation) lysis buffer (50 mM HEPES–KOH at pH 7.5, 140 mM NaCl, 1 mM EDTA, 1% Triton X-100, 0.1% sodium deoxycholate, 0.1% SDS and PI cocktail). Chromatin was sonicated to an average fragment length of 200–500 bp (Bioruptor Pico, Diagenode), diluted with 10 volumes of RIPA buffer (50 mM Tris–HCl at pH 8.0, 150 mM NaCl, 2 mM EDTA, 0.5% sodium deoxycholate, 0.1% SDS and PI cocktail), and incubated with 5 μg of antibodies overnight at 4°C and the incubation was continued with ChIP Grade Protein A/G Plus Agarose (Thermo Fisher Scientific) for 2 h. Then, the beads were washed once with low-salt buffer (150 mM NaCl, 20 mM Tris–HCl at pH 8.0, 1% Triton X-100, 2 mM EDTA, and 0.1% SDS), followed by high-salt buffer containing 500 mM NaCl and LiCl buffer (250 mM LiCl, 10 mM Tris–HCl at pH 8.0, 1 mM EDTA, 1% sodium deoxycholate, 1% NP-40). After elution, reversal of the cross-links (65°C), and digestion with proteinase K, DNA was purified and quantified by qPCR using SYBR Green Master Mix on CFX Connect Real-Time PCR Detection System (Biorad).

### Cell viability assay

Dose-response curves were generated for each cell line by plating 5 × 10^3^/well into a 96-well plate, culturing them for 24 h, and then treating them with the indicated concentrations of ATR inhibitor or RECQ5 inhibitor for 96 h. Cell viability was measured using the CellTiter-Glo assays (Promega) according to the manufacturer’s instructions. Briefly after incubation, room temperature CellTiter-Glo reagent was added 1:1 to each well and the plates were incubated at room temperature for 10 min. Luminescence was measured with the Varioskan LUX Microplate Reader (Thermo Fisher Scientific).

### Annexin-V assay

Apoptotic cell death measurement was performed using FITC Annexin-V Apoptosis Detection Kit (BD Biosciences) as described by the manufacturer and analyzed using flow cytometric analysis on CytoFLEX LX-6L (Beckman).

### Statistical analysis

All results were analyzed using GraphPad Prism 5.0 software. The representative images of IF staining and western blotting are shown. The data are shown as means ± S.D. Significant differences between the two groups were performed using two-tailed Student’s *t*-test.

## Results

### RECQ5 localizes in the nucleolus and is preferentially enriched at DFC

**Figure 1.**
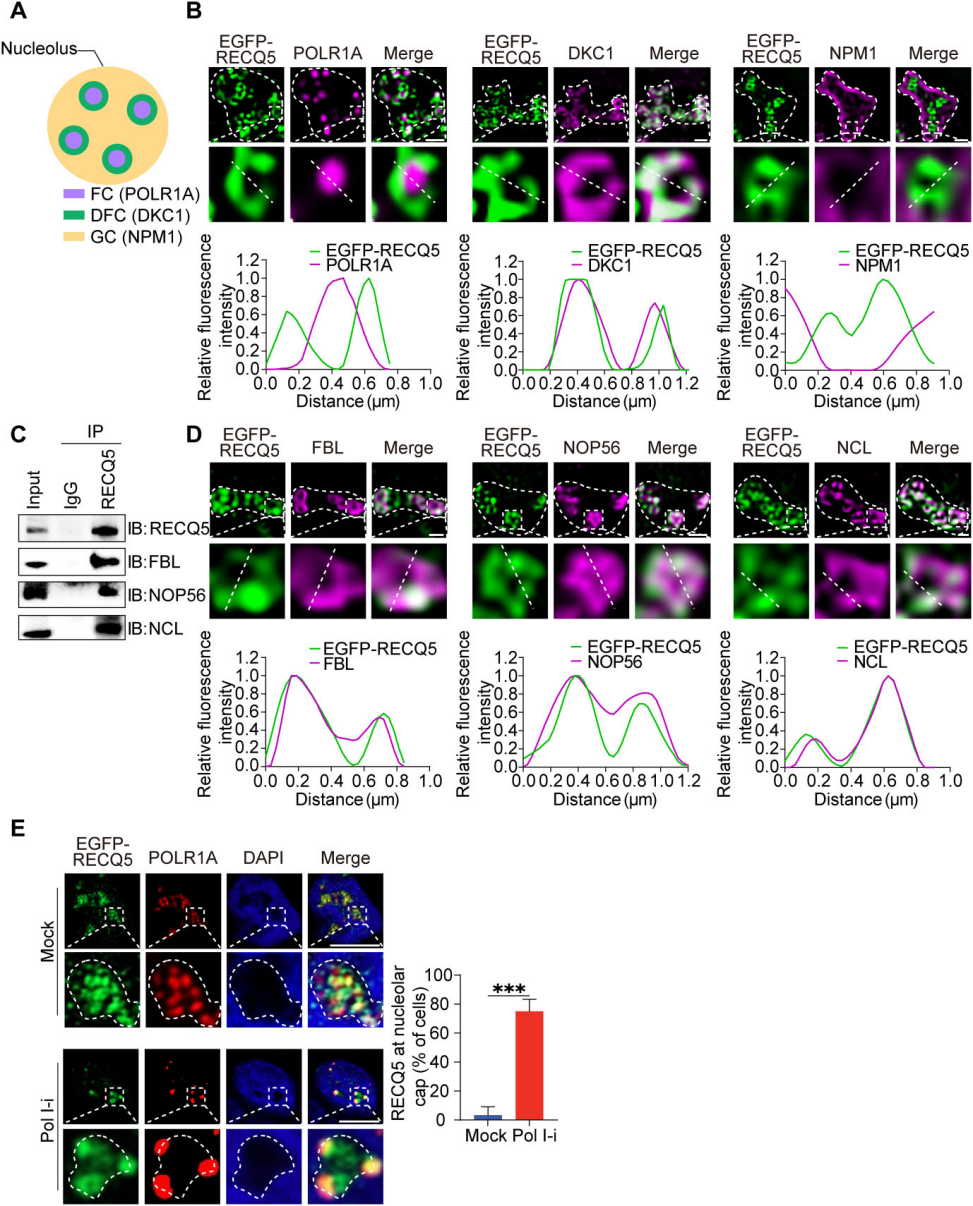
RECQ5 localizes in DFC. (**A**) A schematic diagram shows the three layers of nucleolus. POLR1A, DKC1, and NPM1 are markers for FC, DFC, and GC, respectively. (**B**) EGFP–RECQ5 is enriched in DFC. EGFP–RECQ5 was expressed in U2OS cells. EGFP–RECQ5 and nucleolar markers of FC, DFC, and GC were examined in nucleolus by SIM. The fluorescence intensity on the white dash line was plotted (lower panels). Scale bar, 1 μm. (C, D) RECQ5 interacts and colocalizes with proteins in DFC. Co-immunoprecipitation (Co-IP) and western blotting were performed with indicated antibodies (**C**). EGFP–RECQ5 and DFC-located proteins were examined in nucleolus by SIM. The fluorescence intensity on the white dash line was plotted (lower panels). Scale bar, 1 μm (**D**). (**E**) RECQ5 relocates to nucleolar caps upon inhibition of RNA Pol I. U2OS cells were treated with 1 μM BMH-21 for 2 h. RECQ5 and POLR1A were examined by IF. The percentage of the cells with RECQ5 staining in nucleolar caps was calculated. ***P < .001. Scale bar, 10 μm.

To explore the subcellular localization of endogenous RECQ5 in human cells, we performed IF staining in U2OS cells using antibodies against RECQ5. Interestingly, we found that endogenous RECQ5 displayed an apparent localization in the nucleolus (Supplementary Fig. S1A). Nucleolus is a nuclear condensate comprised of three distinct layers including fibrillar center (FC) where pre-rRNA is transcribed from rDNA loci, dense fibrillar component (DFC) where pre-rRNA is extensively processed, and granular component (GC) where pre-rRNA is associated with protein subunits for pre-rRNP assembly (Fig. 1A) [27, 28]. Using SIM, we examined the detailed localization of RECQ5 inside of nucleolus with POLR1A, DKC1, and NPM1 as markers for FC, DFC, and GC, respectively. We found that RECQ5 mainly colocalized with DKC1, suggesting that RECQ5 exists in DFC (Fig. 1B and Supplementary Fig. S1B).

Moreover, proteomics analyses reveal that a number of nucleolar proteins are associated with RECQ5 (Supplementary Fig. S1C and Supplementary Table S1). We selected three proteins localized in DFC, namely FBL, NOP56, and NCL. Using co-IP and reciprocal IP, we confirmed that RECQ5 interacted with these proteins (Fig. 1C and Supplementary Fig. S1D). Moreover, the interactions were abolished when the cell lysates were treated with RNase but not DNase, suggesting that these interactions are mediated by RNA (Supplementary Fig. S1E). We also examined the detailed localization of RECQ5 with the three nucleolar proteins in DFC. We found that FBL, NOP56, and NCL all colocalized with RECQ5 (Fig. 1D). Thus, the results further demonstrate the localization of RECQ5 in DFC.

In addition, inhibition of Pol I-mediated transcription by BMH-21 induces nucleolar stress and relocation of processing factors from DFC to the cap region of the nucleolus [29]. Similarly, RECQ5 also relocated to the cap region under nucleolar stress (Fig. 1E). Collectively, these results demonstrate that RECQ5 exists in the DFC of the nucleolus.

To further investigate the regulation of the nucleolar localization of RECQ5, we employed a series of distinct RECQ5 regions fused to EGFP and examined their subcellular localization (Supplementary Fig. S2A-C). While the full-length RECQ5–EGFP displayed prominent nucleolar accumulation, the N-terminal mutant with the helicase and RQC domains together (1–453 aa) showed cytoplasmic localization due to the loss of nuclear localization signal (NLS) at the C-terminal end [30]. In contrast, the C-terminal fragment (453–991 aa) exhibited diffuse nuclear staining with exclusion from the nucleolus. Interestingly, when the N-terminal region (1–453 aa) was fused to the NLS, it conferred clear nucleolar localization, indicating that the NoLS (Nucleolar localization sequence) is present in the N-terminal region (1–453 aa).

To further delineate the minimal nucleolar targeting sequence, we generated several N-terminal deletion mutants fused to an NLS. Both Δ1-338 aa and Δ339-379 aa mutants retained a clear pattern of nucleolar enrichment, whereas deletion of residues 380–453 resulted in diffuse nuclear distribution with nucleolar exclusion. Strikingly, a minimal construct spanning residues 380–453 alone exhibited nucleolar localization when coupled with an NLS. Collectively, these findings demonstrate that residues 380–453 of RECQ5 constitute a NoLS.

### RECQ5 plays an important role in pre-rRNA processing

**Figure 2.**
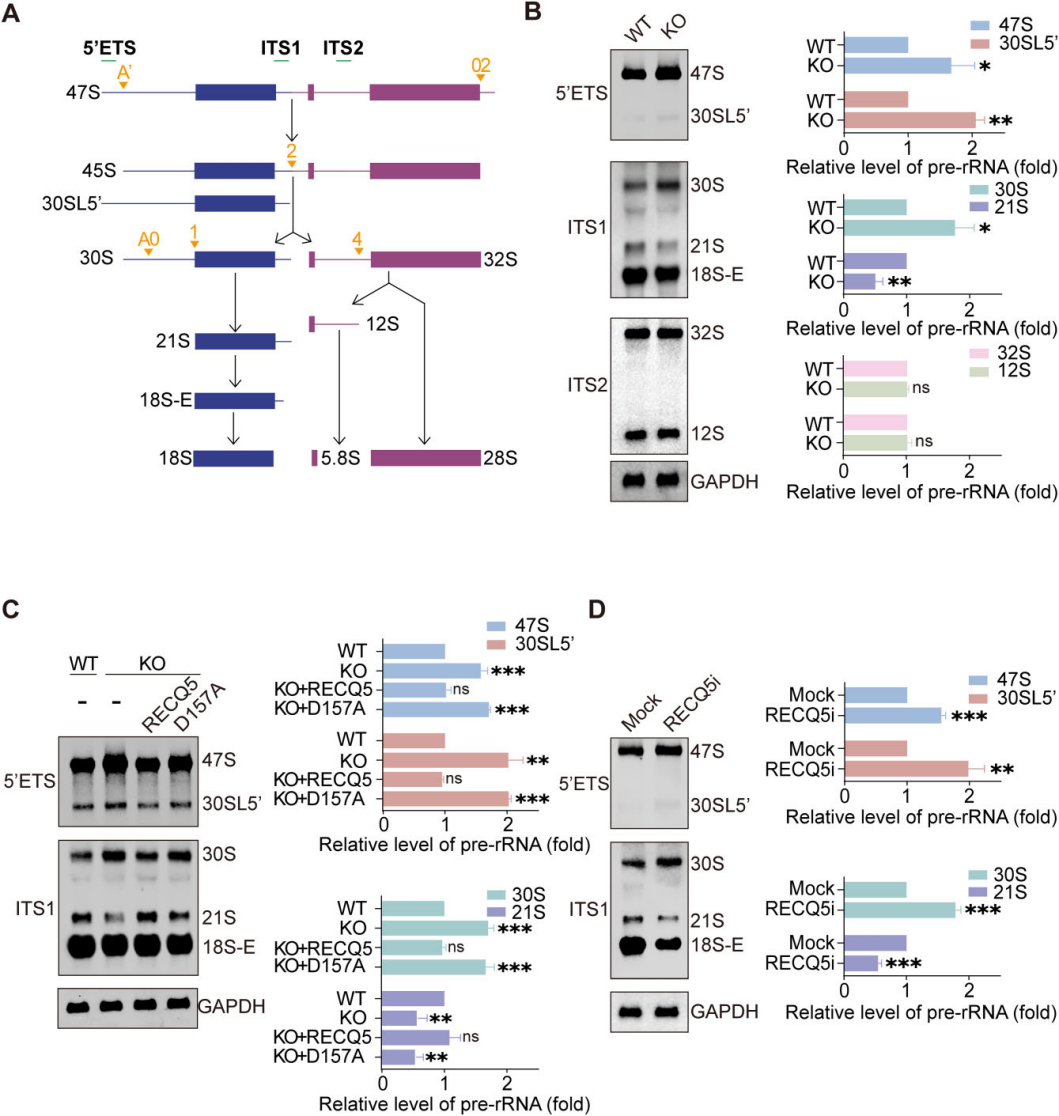
Loss of RECQ5 impairs pre-rRNA processing. (**A**) Schematic view of the major pre-rRNA precursors detected in human cells. The probes targeting 5′ETS, ITS1, and ITS2 for northern blotting are indicated by the green lines. The cleavage sites at each processing step are indicated by the yellow marks. (**B**) RECQ5 depletion disrupts pre-rRNA processing. Northern blotting was performed to detect pre-rRNA intermediates in WT and RECQ5-KO HeLa cells. The relative levels of 47S, 30SL5′, 30S, 21S, 32S, and 12S pre-rRNA were measured. GAPDH was used as the loading control. (**C**) RECQ5 regulates pre-rRNA processing, which is dependent on the helicase activity. Northern blotting was performed in cells deleted endogenous RECQ5 and reexpressing ectopic RECQ5, helicase-dead mutant RECQ5^D157A^. The relative levels of 47S, 30SL5′, 30S, and 21S pre-rRNA were measured. (**D**) RECQ5 inhibitor treatment impairs pre-rRNA processing. HeLa cells were treated with RECQ5-IN-1 (5 μM) for 24 h. Northern blotting was performed to detect pre-rRNA intermediates. The relative levels of 47S, 30SL5′, 30S, and 21S pre-rRNA were measured. Data are represented as means ± SD as indicated. Two-tailed Student’s *t*-test was used to determine statistical significance. *P < .05; **P < .01; ***P < .001; ns, not significant.

Following the Pol I-mediated transcription of 47S pre-rRNA from rDNA loci, the pre-rRNA undergoes several steps of processing in DFC [27]. 47S pre-rRNA is processed to 45S, which is further digested into 30S and 32S intermediates. 30S pre-rRNA is processed into 21S that is converted into 18S-E, the precursor of 18S ribosomal RNA (rRNA). 32S pre-rRNA encompasses several cleavage reactions and is digested into 5.8S and 28S rRNA (Fig. 2A). To examine the biological function of RECQ5 in pre-rRNA processing, we generated RECQ5 knockout (RECQ5-KO) HeLa cells using the CRISPR–Cas9 system and validated with anti-RECQ5 antibody (Supplementary Fig. S3A and B). Using 5-EU incorporation assays, we did not observe obvious defects of RNA synthesis in the RECQ5-KO cells (Supplementary Fig. S3C). Since >80% of nascent RNA is pre-rRNA, these results suggest that loss of RECQ5 does not obviously affect pre-rRNA transcription.

Next, we performed northern blotting assays with various probes hybridizing to 5′ETS, ITS1, and ITS2 to examine the intermediates of pre-rRNA (Fig. 2A). The readout obtained by northern blotting assays possesses adequate linearity to permit quantitative evaluation (Supplementary Fig. S4A). Interestingly, we observed that lacking RECQ5 induced an obvious 30S accumulation and a clear reduction of 21S pre-rRNA, indicating that RECQ5 plays an important role during 30S pre-rRNA being processed to 21S pre-rRNA (Fig. 2B and Supplementary Figs S4B and S5B). Moreover, with a probe targeting 5′ETS, the results show that 47S and 30SL5′ (5′ extended version of 30S) were also accumulated in cells lacking RECQ5 (Fig. 2B and Supplementary Figs S4B and S5B), indicating that A′ site cleavage is also suppressed in these cells. Since the level of 47S pre-rRNA can also be affected by Pol I-mediated transcription, we treated RECQ5 WT and KO cells with 5-FU to abrogate the processing of pre-rRNA, and then performed pulse labeling with 4sU to detect newly synthesized 47S pre-rRNA [24]. Compared to that in the wild-type cells, the newly synthesized 47S pre-rRNA in RECQ5-KO cells was not affected, suggesting that RECQ5 regulates 47S pre-rRNA not at the transcriptional level (Supplementary Fig. S6). Collectively, these results suggest that loss of RECQ5 impairs the processing of 5′ETS.

To investigate the role of RECQ5 helicase activity in pre-rRNA processing, we mutated Asp157 to Ala (D157A) to abolish the ATPase activity. Consistently, 47S, 30SL5′, and 30S pre-rRNAs were accumulated, while 21S pre-rRNA was reduced (Fig. 2C and Supplementary Figs S4C and S5C). Of note, the D157A mutation did not affect the nucleolar localization of RECQ5 (Supplementary Fig. S7A and B). Moreover, to validate the results, we treated HeLa cells with RECQ5-IN-1, a highly selective RECQ5 inhibitor [31]. Again, the cleavage at A′ and the transition from 30S to 21S were impaired (Fig. 2D and Supplementary Figs S4D and S5D). Taken together, these results suggest that RECQ5 facilitates pre-rRNA processing through its helicase activity.

### RECQ5 unwinds dsRNA *in vitro*

**Figure 3.**
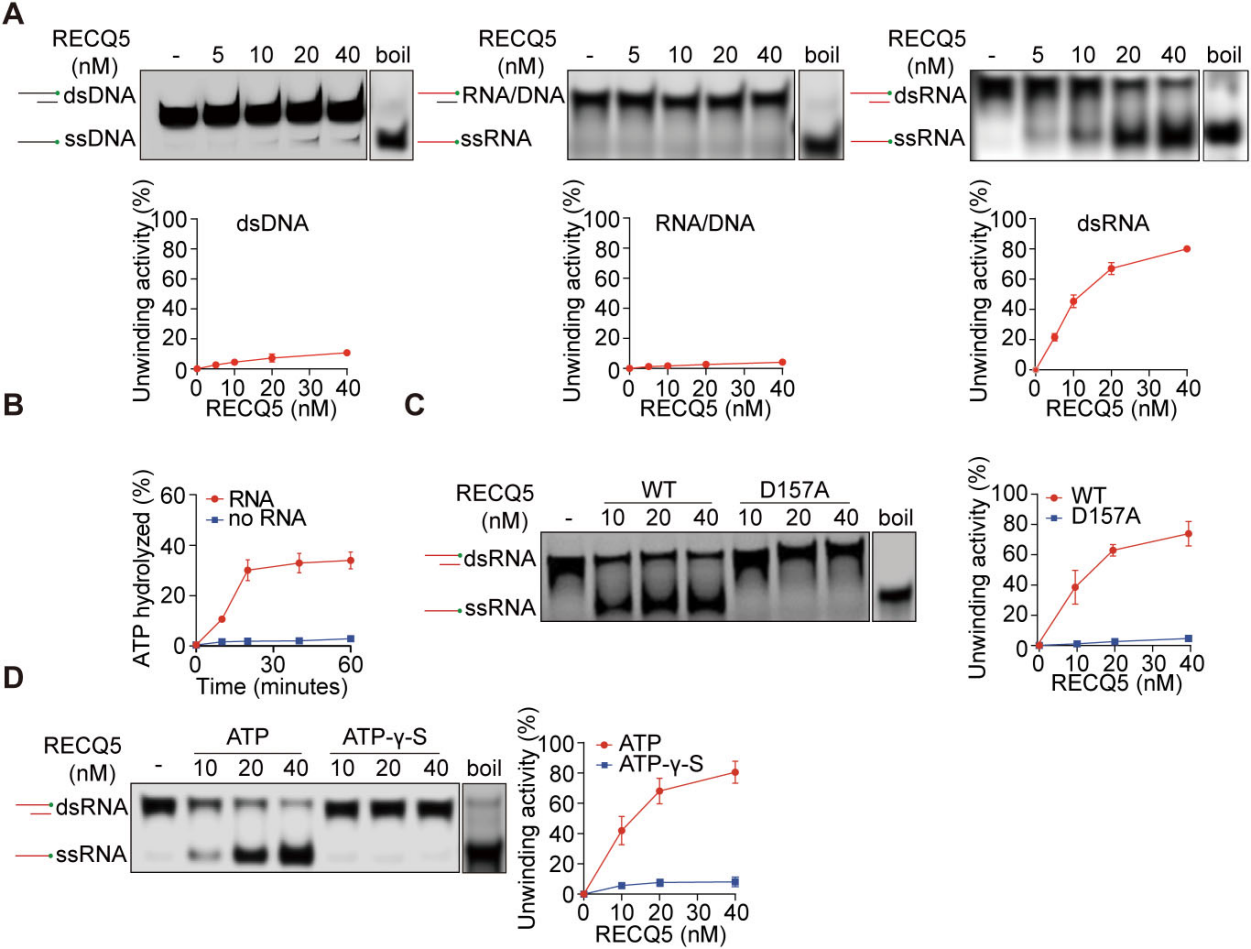
RECQ5 unwinds dsRNA *in vitro*. (**A**) RECQ5 unwinds the dsRNA. Recombinant RECQ5 was incubated with dsDNA, RNA/DNA hybrids, and dsRNA. The reaction products were analyzed using gel electrophoresis. (**B**) RECQ5 consumes ATP in the presence of RNA. Recombinant RECQ5 was incubated with or without dsRNA. The ATP levels were measured. (**C**) The D157A mutation abolishes the enzymatic activity of RECQ5. The recombinant D157A mutant was generated from *E. coli*. (**D**) The helicase activity of RECQ5 is dependent on ATP. ATP or adenosine 5-[γ-thio] triphosphate (ATP-γ-S) was included in the *in vitro* helicase assays.

It has been previously established that helicase facilitates pre-rRNA processing by unwinding pre-rRNA secondary structures [32, 33]. To understand how RECQ5 controls pre-rRNA processing, we investigated the helicase activity of RECQ5 using *in vitro* helicase assays with BLM and DDX3X as positive controls for DNA and RNA duplex unwinding (Supplementary Fig. S8A and B) [34, 35]. We purified the recombinant RECQ5 and incubated the protein with different nucleic acid substrates (Supplementary Fig. S8C). Surprisingly, RECQ5 had a very weak helicase activity to unwind double-stranded DNA (dsDNA) or the RNA/DNA hybrid (Fig. 3A and Supplementary Fig. S8D). However, RECQ5 could efficiently unwind the double-stranded RNA (dsRNA) (Fig. 3A). The amount of unwound single-stranded RNA was increased along with the increased amount of RECQ5, suggesting that this unwinding process is mediated by RECQ5. Moreover, we examined RNA duplexes with various overhang tail lengths from 0 to 35 nt. The results reveal that RECQ5 requires an overhang with at least 15 nt for efficient dsRNA unwinding (Supplementary Fig. S8E), indicating that RECQ5 might unwind RNA by a translocation mechanism, and the ssRNA overhang may serve as a loading dock for RECQ5 during unwinding. We also examined the unwinding activity with different lengths of RNA duplexes and found that the unwinding activity of RECQ5 was reduced on longer duplexes (Supplementary Fig. S8F), indicating that RECQ5 may only efficiently unwind shorter RNA duplexes, which often exist in the higher-order structure of RNA species. In addition, RECQ5 unwinds dsRNA in a sequence-independent manner, consistent with the notion that RNA helicases are often considered nonspecific for RNA (Supplementary Fig. S8G) [36].

Next, we measured the ATPase activity of RECQ5 at a fixed protein concentration of 10 nM. Along with the increased incubation time, ATP could be consumed by RECQ5 in the presence of dsRNA substrates but not dsDNA (Fig. 3B and Supplementary Fig. S8H). Moreover, the D157A mutant that abolishes the ATPase activity of RECQ5 failed to unwind dsRNA, suggesting that the catalytic activity of RECQ5 is required for dsRNA resolution (Fig. 3C and Supplementary Fig. S8I). To further validate the results, we used the helicase inhibitor to suppress the ATPase activity of RECQ5. However, RECQ5-IN-1 only partially suppresses the ATPase activity [31]. Thus, we used ATP-γ-S, a non-hydrolysable ATP analogue, to measure the helicase activity of RECQ5 in the assay, and RECQ5 was no longer able to unwind dsRNA, further demonstrating that RECQ5 consumes ATP for dsRNA unwinding (Fig. 3D). Collectively, RECQ5 is an RNA helicase that can unwind dsRNA into ssRNA, which may play an important role in secondary structure unwinding during pre-rRNA processing.

### Cancer-associated mutations of RECQ5 abolish its nucleolar localization or the helicase activity

**Figure 4.**
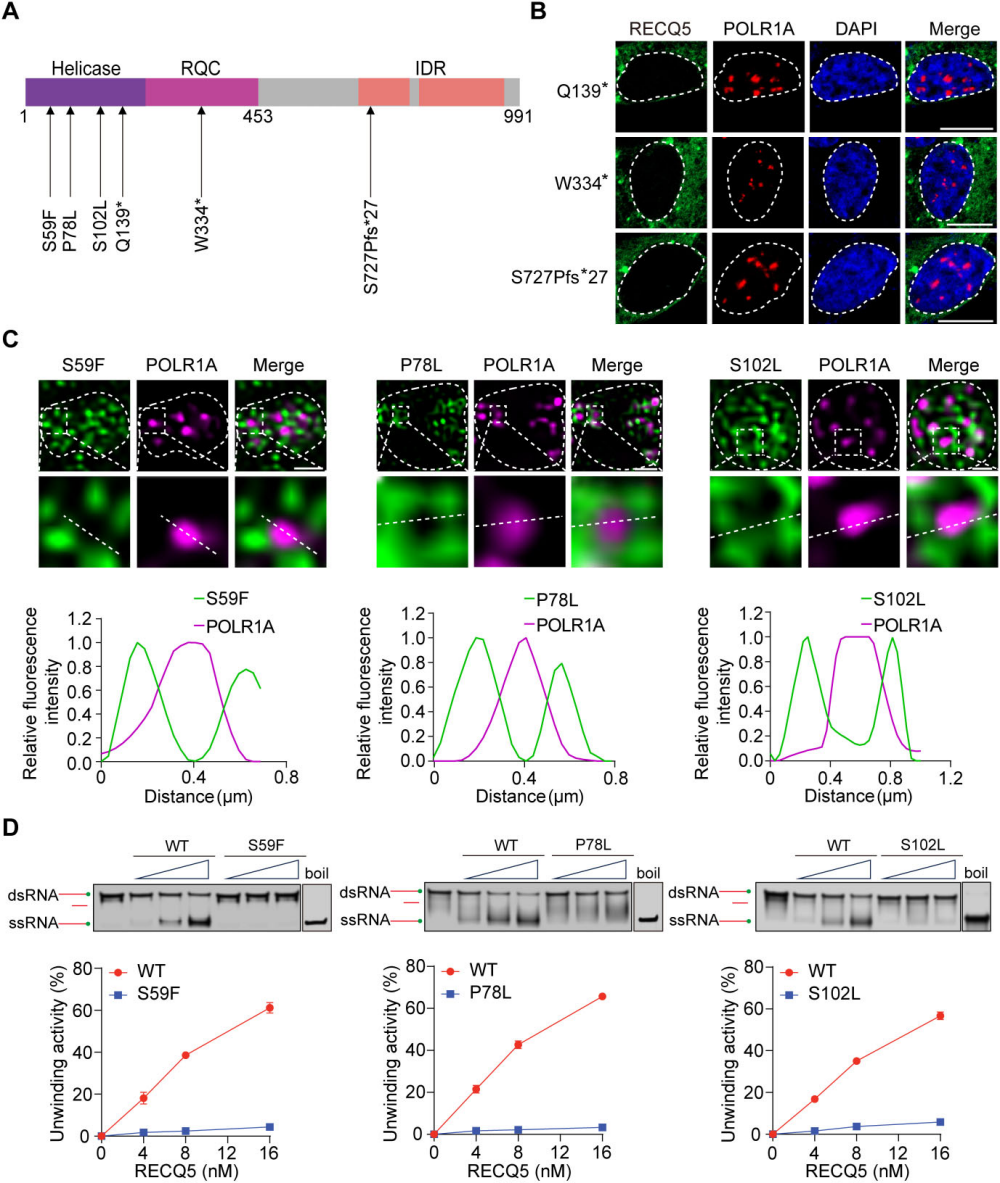
Characterization of cancer-associated mutations of RECQ5. (**A**) Schematic of the representative mutations of RECQ5. (**B**) The truncation mutants, including Q139*, W334*, and S727Pfs*27, do not localize in nucleolus. GFP-tagged RECQ5 mutants and POLR1A were examined by IF. Scale bar, 10 μm. (**C**) The missense mutations, including S59F, P78L, and S102L, do not affect the nucleolar localization of RECQ5. The GFP-tagged RECQ5 mutants and POLR1A were examined in nucleolus by SIM. The fluorescence intensity on the white dash line was plotted (lower panels). (**D**) The missense mutations impair the helicase activities of RECQ5. Wild-type RECQ5 or RECQ5 mutants were incubated with the dsRNA substrates, and *in vitro* helicase assays were conducted. The reaction products were analyzed using gel electrophoresis (lower panels).

Both germline and spontaneous mutations of RECQ5 have been identified in various types of cancer [2, 13]. From the TCGA and COSMIC databases, we have summarized these cancer-associated mutations into three groups, namely missense mutation, nonsense mutation, and frameshift mutation. First, we examined three truncation mutations, Q139*, W334*, and S727Pfs*27, caused by nonsense mutations or frameshift mutation. These representative mutations abolish the helicase domain, the RQC domain, and the C-terminal disordered region, respectively (Fig. 4A and Supplementary Table S2). When these mutants were expressed in HeLa cells, all of them were excluded from the nucleus, because these deletions abolish the NLS at the C-terminus (Fig. 4B and Supplementary Figs S2 and S9).

Since the major function of RECQ5 is to unwind dsRNA, we also examined three representative missense mutations in the helicase domain of RECQ5. Unlike the truncation mutations, the missense mutations, including S59F, P78L, and S102L, did not affect the nucleolar localization of RECQ5 and still displayed a ring structure surrounding POLR1A (Fig. 4C and Supplementary Fig. S9). However, we found that the helicase activities of the three mutants were disrupted (Fig. 4D). The structural analyses indicate that S59 localizes in the catalytic pocket of the ATPase domain with the side chain coordinating with ATP. The S59F mutation likely abolishes the ATPase pocket (Supplementary Fig. S10A). Both Pro78 and Ser102 localize at the putative RNA-binding surface. The P78L mutation may cause a conformational change in the whole region to disrupt the helicase activity (Supplementary Fig. S10B). This conformation maintains the helicase activity of RECQ5 under normal conditions [4]. Moreover, the side chain of Ser102 may directly contact the RNA substrate, and the S102L mutation may disrupt the substrate binding (Supplementary Fig. S10C). Collectively, these cancer-associated mutations disrupt either the proper localization or enzymatic activity of RECQ5 and likely act as pathogenic mutations driving genomic instability and tumorigenesis.

### Loss of RECQ5 induces RNA/DNA hybrids and DNA damage

**Figure 5.**
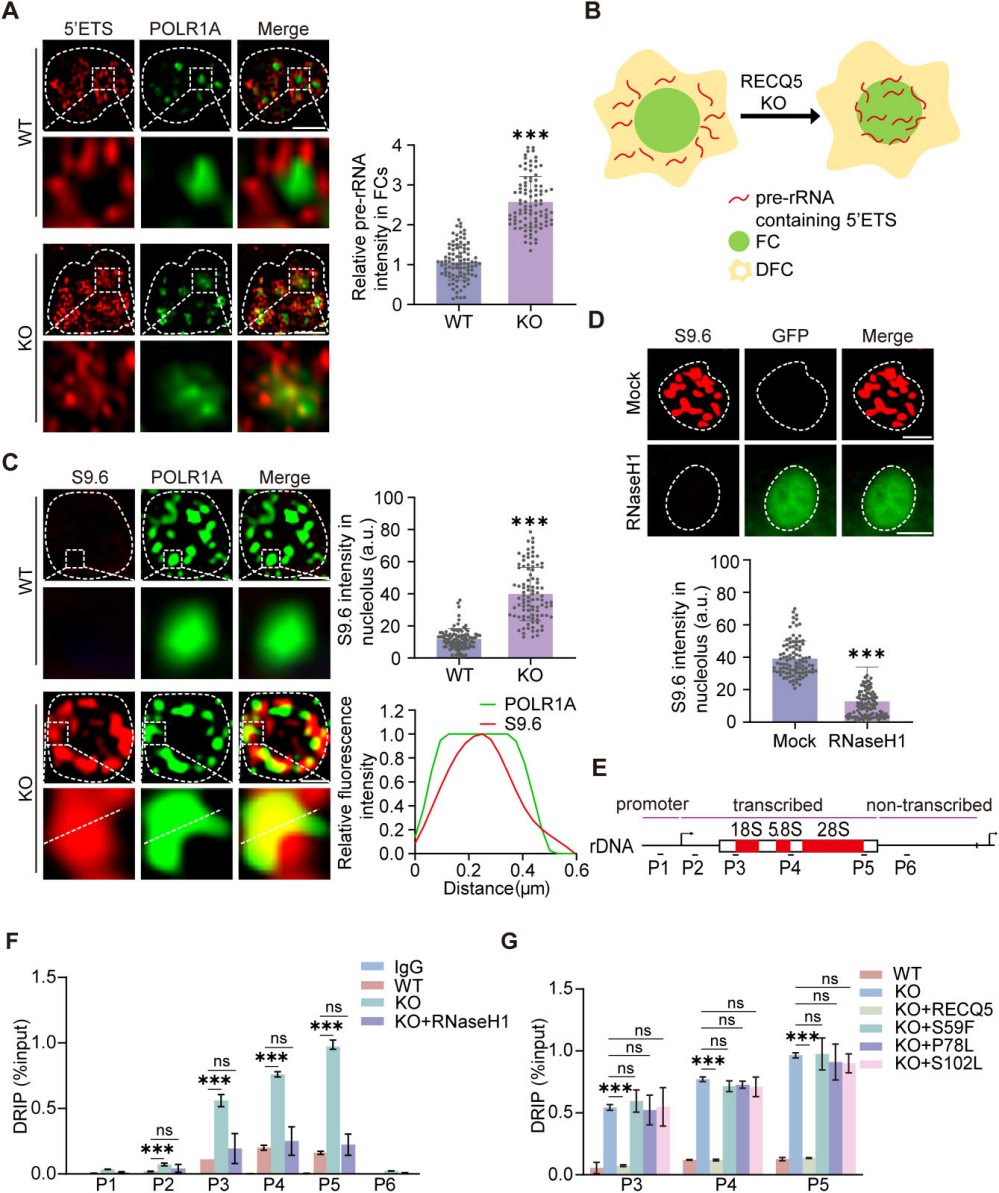
Loss of RECQ5 induces the RNA/DNA hybrid at rDNA loci. (**A**) Loss of RECQ5 leads to the accumulation of unprocessed pre-rRNA in FC. Nascent pre-rRNA was detected in nucleolus by 5′ETS probe in cells lacking RECQ5. SIM was performed. The staining of POLR1A indicates FC. Scale bars, 1 μm. The relative signal intensity of 5′ETS was calculated in 100 FC (right panel). (**B**) A model of RECQ5-mediated pre-rRNA releasing from FC. (**C**) Loss of RECQ5 induces accumulation of the RNA/DNA hybrid in nucleolus. The RNA/DNA hybrids were stained in nucleolus by S9.6 antibody. The staining of POLR1A indicates FC. SIM was performed. 100 nucleoli were examined in each group. Scale bars, 1 μm. The fluorescence intensity on the white dash line was plotted (lower right panel). (**D**) Ectopically expressed RNaseH1 suppresses the RNA/DNA hybrid. RNaseH1-GFP and empty vector were expressed in HeLa cells lacking RECQ5. The RNA/DNA hybrid was examined in nucleolus by S9.6. 100 nucleoli were examined in each group. Scale bars, 1 μm. (**E**) The positions of primer pairs at rDNA locus. (**F**) Loss of RECQ5 causes the RNA/DNA hybrid at rDNA locus. DRIP-qPCR was performed with S9.6 antibody and indicated primers. The assays were triplicated. (**G**) Wild-type but not cancer-associated mutations of RECQ5 suppress the RNA/DNA hybrid at rDNA locus. Data are represented as means ± SD as indicated. Two-tailed Student’s *t*-test was used to determine statistical significance. ***P < .001; ns, not significant.

Loss of RECQ5 is associated with genomic instability [37, 38]. However, the molecular mechanism underlying this phenomenon is still unclear. It has been previously found that mutants defective in RNA processing factors exhibit increased genome instability [39]. Given that RECQ5 mediates pre-rRNA processing at 5′ETS segment (Fig. 2), we ask whether RECQ5 maintains genomic stability at rDNA loci. Thus, we used probes targeting 5′ETS to observe the nascent pre-rRNAs. Interestingly, we observed that in the wild-type cells, the nascent Cy3-labeled pre-rRNA containing 5′ETS segment displayed a ring-like structure and localized surrounding FC (Fig. 5A), suggesting that the transcribed pre-rRNA was released from FC. However, in RECQ5-deficient cells, where 5′ETS processing was suppressed, we noticed that the pre-rRNA containing 5′ETS failed to form the ring-like structure. Instead, it accumulated in FC (Fig. 5A and B). Thus, these results suggest that loss of RECQ5 suppresses 5′ETS processing, leading to the accumulation of unprocessed pre-rRNAs in FC.

rDNA loci in FC are the most highly transcribed regions in cells, suggesting that most rDNA is likely in an unwound state. Thus, we hypothesize that the accumulated unprocessed pre-rRNA in FC is hybridized with rDNA. Consistently, using the S9.6 antibody to monitor the RNA/DNA hybrid, we found that the level of RNA/DNA hybrid was drastically elevated in the RECQ5-deficient cells and mainly accumulated in FC (Fig. 5C). Since RNaseH1 is an enzyme that specifically removes RNA from the RNA/DNA hybrid [40], we expressed GFP-RNaseH1 in the RECQ5-deficient cells and found that the signals of S9.6 staining were remarkably reduced, further suggesting that loss of RECQ5 induces the RNA/DNA hybrid in the nucleolus (Fig. 5D).

Using DRIP-qPCR, we also confirmed that the RNA/DNA hybrid accumulated at rDNA loci when cells lacked RECQ5, and ectopically expressed RNaseH1 reduced the levels of the RNA/DNA hybrid in these cells (Fig. 5E and F, and Supplementary Fig. S11A). Moreover, reintroducing wild-type RECQ5 but not those cancer-associated mutants were able to suppress the RNA/DNA hybrid at rDNA loci (Fig. 5G and Supplementary Fig. S11B), indicating that the RNA helicase activity of RECQ5 inhibits the RNA/DNA hybrid accumulation. Taken together, these results suggest that loss of RECQ5 hinders the processing of 5′ETS, resulting in the accumulation of unprocessed pre-rRNAs in FC, which causes an increase in RNA/DNA hybrids.

Since excessive RNA/DNA hybrid causes DNA damage and subsequent genome instability [41], we examined γH2AX, a surrogate marker for DSBs. Again, in the RECQ5-deficient cells, the level of γH2AX was clearly increased at rDNA loci, and it was removed when ectopic RNaseH1 was expressed (Supplementary Fig. S12). Collectively, these results show that loss of RECQ5 induces the RNA/DNA hybrid at rDNA loci, which causes DNA damage.

### Loss of RECQ5 induces ATR activation and sensitizes tumor cells to ATR inhibitor treatment

**Figure 6.**
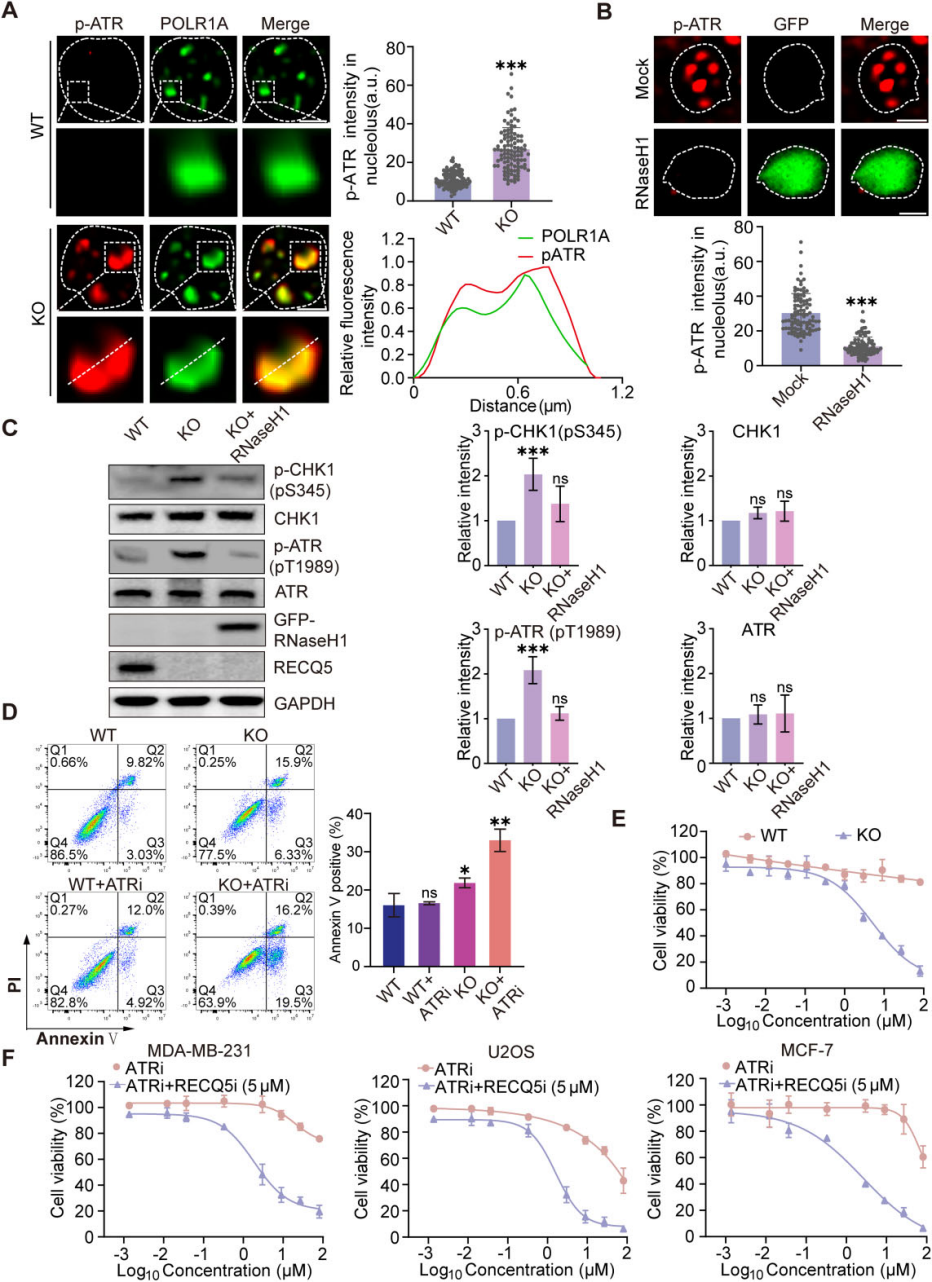
Loss of RECQ5 induces cancer cell lines hypersensitive to ATR inhibitor treatment. (**A**) Loss of RECQ5 induces ATR activation in nucleolus. pATR (pT1989) was examined in nucleolus. The staining of POLR1A indicates FC. SIM was performed. 100 nucleoli were examined in each group. Scale bars, 1 μm. The fluorescence intensity on the white dash line was plotted (lower right panel). (**B**) Ectopically expressed RNaseH1 suppresses ATR activation in the RECQ5-deficient cells. RNaseH1-GFP and empty vector were expressed in HeLa cells lacking RECQ5. pATR was examined in nucleolus. 100 nucleoli were examined in each group. Scale bars, 1 μm. (**C**) Loss of RECQ5 activates ATR-dependent signal pathway. Western blotting was performed with indicated antibodies. GAPDH was used as a protein loading control. The quantitative analysis (relative to GAPDH) was performed. (**D**) ATR inhibitor treatment induces apoptosis in RECQ5-deficient cells. The apoptotic cells were examined by flow cytometry with staining of Annexin-V and PI. *n* = 3. (**E**) RECQ5-deficient cells are hypersensitive to ATR inhibitor (VE-821) treatment. RECQ5-WT and RECQ5-KO cells were treated with the indicated dose of VE-821 for 4 days. Cell viability was determined using CellTiter-Glo assays. (**F**) Combination treatment with RECQ5i (RECQ5-IN-1) and ATRi (VE-821) suppresses the growth of cancer cell lines. MDA-MB-231, U2OS, and MCF-7 cells were treated with the indicated dose of ATRi or together with 5 μM of RECQ5i for 4 days. Data are represented as means ± SD as indicated from three independent experiments. Two-tailed Student’s *t*-test was used to determine statistical significance. *P < .05; **P < .01; ***P < .001; ns, not significant.

It has been shown that ATR is a key sensor of R-loops and suppresses R-loop-induced genomic instability [42, 43]. Once ATR is activated, it autophosphorylates itself [44]. Thus, phospho-ATR (pATR) is a surrogate marker for the ATR activation. As expected, we observed pATR foci at FC of nucleolus in the RECQ5-KO cells (Fig. 6A). Again, when we expressed RNaseH1 to remove the RNA/DNA hybrid, the pATR foci formation in nucleoli was abolished (Fig. 6B). Moreover, ATR activation also induces phosphorylation of downstream CHK1 at Ser345 [45, 46]. Again, with western blotting, we found the upregulation of pSer345 of CHK1 and pT1989 of ATR in the RECQ5-deficient cells and demonstrated that RECQ5 depletion-induced ATR phosphorylation is primarily dependent on TOPBP1 due to R loop accumulation (Fig. 6C and Supplementary Fig. S13) [43]. Additionally, APE1 plays a minor role, possibly due to its recruitment in this process and its capacity to act as a regulator to directly activate ATR (Supplementary Fig. S13) [47].

Activation of ATR induces DNA damage response including cell cycle checkpoint activation and DNA damage repair [48, 49]. In contrast, suppression of ATR activation aggravates DNA damage and induces cell apoptosis. Thus, ATR inhibitors have been developed for the treatment of tumor cells with DNA damage repair defects [50]. Consistently, we found that compared to the RECQ5-proficient cells, the RECQ5-deficient cells were hypersensitive to ATR inhibitor treatment (Fig. 6D and E). Moreover, we randomly tested three cell lines including MDA-MB-231, U2OS, and MCF7, and found that all of them were insensitive to RECQ5 inhibitor treatment (Supplementary Fig. S14). However, RECQ5 inhibitor treatment could sensitize these tumor cells to ATR inhibitor treatment (Fig. 6F). Therefore, RECQ5 inhibitor and ATR inhibitor synergistically induce tumor cell lethality, which may lead to novel therapeutic approaches for cancer treatment.

## Discussion

In this study, we demonstrate that RECQ5 resides in the nucleolus for pre-rRNA processing. Loss of RECQ5 induces accumulation of 47S, 30SL5′, and 30S pre-rRNA, suggesting that RECQ5 regulates the processing of pre-rRNA at A′, A0, and 1 sites. However, RECQ5 itself is not an RNase. Instead, it is an RNA helicase and is able to unwind dsRNA. It is possible that RECQ5 unwinds newly synthesized pre-rRNA and prevents the newly synthesized pre-rRNA from forming any secondary structure, so that 5′ of pre-rRNA can be processed. If cells lose RECQ5, part of the unprocessed nascent pre-rRNA may anneal to the rDNA loci forming R-loops, which induce genomic instability. Similarly, any cancer-associated mutations abolishing the helicase activity of RECQ5 also cause the R-loop formation and ultimately induce genomic instability.

The localization of RECQ5 at the nucleolus provides additional support to explain the function of RECQ5. We demonstrate that a region with amino acids 380–453 mediates the nucleolar localization. This region is a degenerated RQC domain with a Zn^2+^ binding motif and an additional α-helix. This domain has long been inferred to be responsible for binding nucleic acids, due to the cluster of positively charged residues that could form polar contacts to the backbone of nucleic acids [4]. With the SIM imaging, we found that RECQ5 was enriched in DFC surrounding Pol I to form a ring structure, suggesting that once the pre-rRNA is transcribed by Pol I, RECQ5 starts to unwind any secondary structure of nascent pre-rRNA for 5′ processing steps, which allows pre-rRNA release from FC. When RECQ5 was deleted, the RNA/DNA hybrids were formed in FC, suggesting that the nascent pre-rRNA may anneal to rDNA forming R-loops. Consistently, we examined the localization of the 5′ETS of pre-rRNA. In the presence of RECQ5, it was released into DFC for processing. However, in the absence of RECQ5, the 5′ETS of pre-rRNA remains in FC, further suggesting that the defects in the processing of the 5′ETS induced possible R-loop formation between the 5′ETS and rDNA.

In the *in vitro* assay, RECQ5 favors unwinding dsRNA. It is consistent with the localization of RECQ5 in DFC, where only RNA but not rDNA exists in DFC. Thus, under physiologically relevant conditions, RECQ5 may unwind the secondary structure of pre-rRNA, such as hairpins or G4, to facilitate 5′ETS processing. In addition to RECQ5, other RECQ family members, such as BLM, may also localize in nucleolus [51, 52]. Thus, it is possible that other RECQ family members may also participate in pre-rRNA processing. Of note, we also observed that RECQ5 had a weak activity to unwind RNA/DNA hybrids (Supplementary Fig. S8D), suggesting that this activity of RECQ5 may also contribute to removing the R-loops from rDNA.

The R-loop is a source of genome stability, and R-loops accumulate preferentially at highly transcribed genes [53]. Since pre-rRNA transcription is 80%–90% of total RNA transcription, rDNA loci are the highest transcribed regions in the cell. Here, we have shown that RECQ5 is a safeguard to suppress R-loops in the highest transcribed regions. Once the tumor cells harbor mutations to abolish RECQ5, it causes the R-loop formation and triggers the activation of ATR. Suppression of ATR by small molecule inhibitors under this condition induces massive DSBs and cell lethality. Thus, tumor cells lacking RECQ5 are hypersensitive to ATR inhibitor treatment, which may lead to precision treatment on tumors with RECQ5 mutations. Moreover, RECQ5 inhibitors and ATR inhibitors can synergistically kill tumor cells. Thus, any recently developed delivery systems, such as tumor-targeting antibodies, may conjugate these inhibitors for future tumor suppression.

## Supplementary Material

gkaf766_Supplemental_Files

## Data Availability

All datasets have been deposited in the ProteomeXchange Consortium via the iProX repository with the dataset identifier PXD058022.
