# Supplementary material for: RECQ5 mediates pre-rRNA processing in nucleolus
Source: Nucleic Acids Res. 2025 Aug 18;53(15):gkaf766. doi: 10.1093/nar/gkaf766 (PMC12359038; doi:10.1093/nar/gkaf766)
Supplement: gkaf766_Supplemental_Files [file gkaf766_supplemental_files.zip › Supplemental Figures.pdf]

Supplemental Information

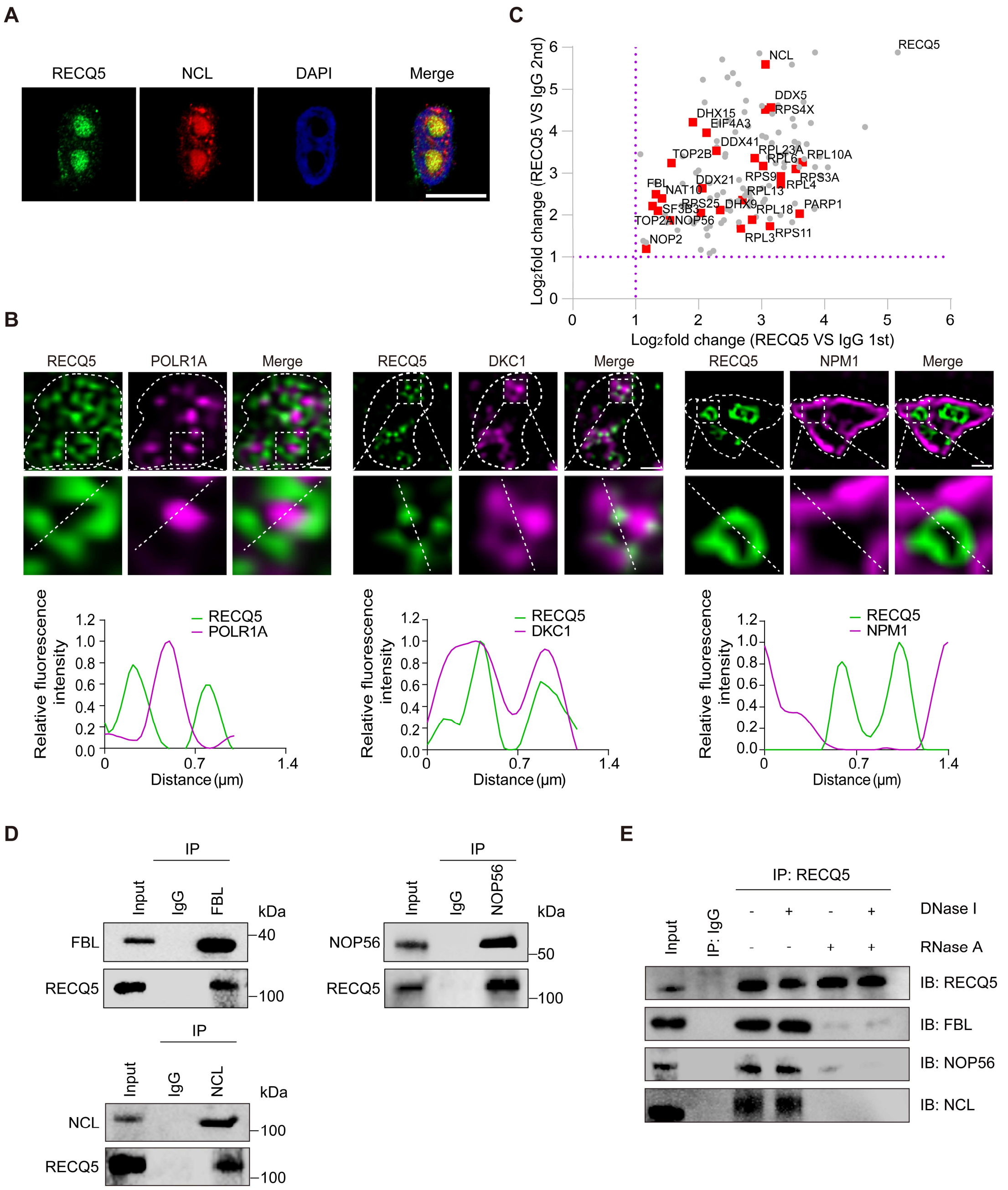

Supplemental Figure S1. RECQ5 localizes in nucleolus and interacts with proteins of DFC.

(A) Endogenous RECQ5 is enriched in nucleolus. RECQ5 was examined by IF in U2OS cells. NCL (nucleolin) is used as a marker for nucleolus. Scale bar, 10 μm. **(B)** Endogenous RECQ5 is enriched in DFC. Endogenous RECQ5 and nucleolar markers

of FC, DFC and GC were examined in nucleolus of U2OS cells by SIM. The fluorescence intensity on the white dash line was plotted (lower panels). Scale bar, 1 μm. **(C)** RECQ5-associated proteins. RECQ5-associated proteins were examined by quantitative mass spectrometry. Results from duplicated assays are shown. The red squares represent proteins known in the nucleolus. **(D)** FBL, NOP56 and NCL are associated with RECQ5. Western blotting was performed with the indicated antibodies. **(E)** RECQ5 interacts with proteins of DFC. Co-IP analysis of the association of RECQ5 with proteins in DFC in the absence or presence of different nucleases as indicated.

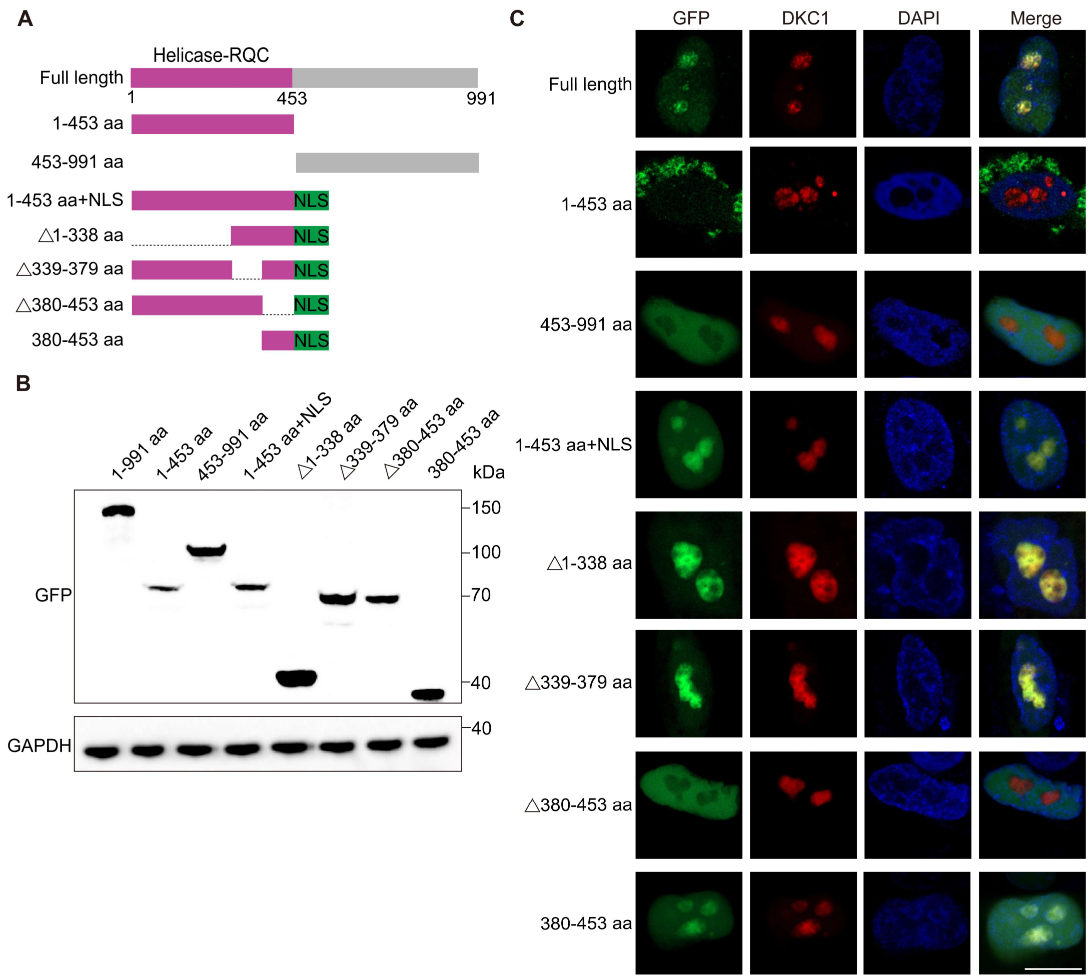

Supplemental Figure S2. Subcellular localization and expression of RECQ5 mutants.

(A) Schematic diagram of GFP-tagged RECQ5 mutants. **(B)** Protein expression of GFP-tagged RECQ5 mutants. **(C)** Intranuclear localization of GFP-tagged RECQ5 mutants. U2OS cells were transfected with different constructs. The GFP-tagged RECQ5 mutants and DKC1 were examined by a Zeiss LSM 900 Airyscan inverted confocal microscope. Scale bar, 10 μm.

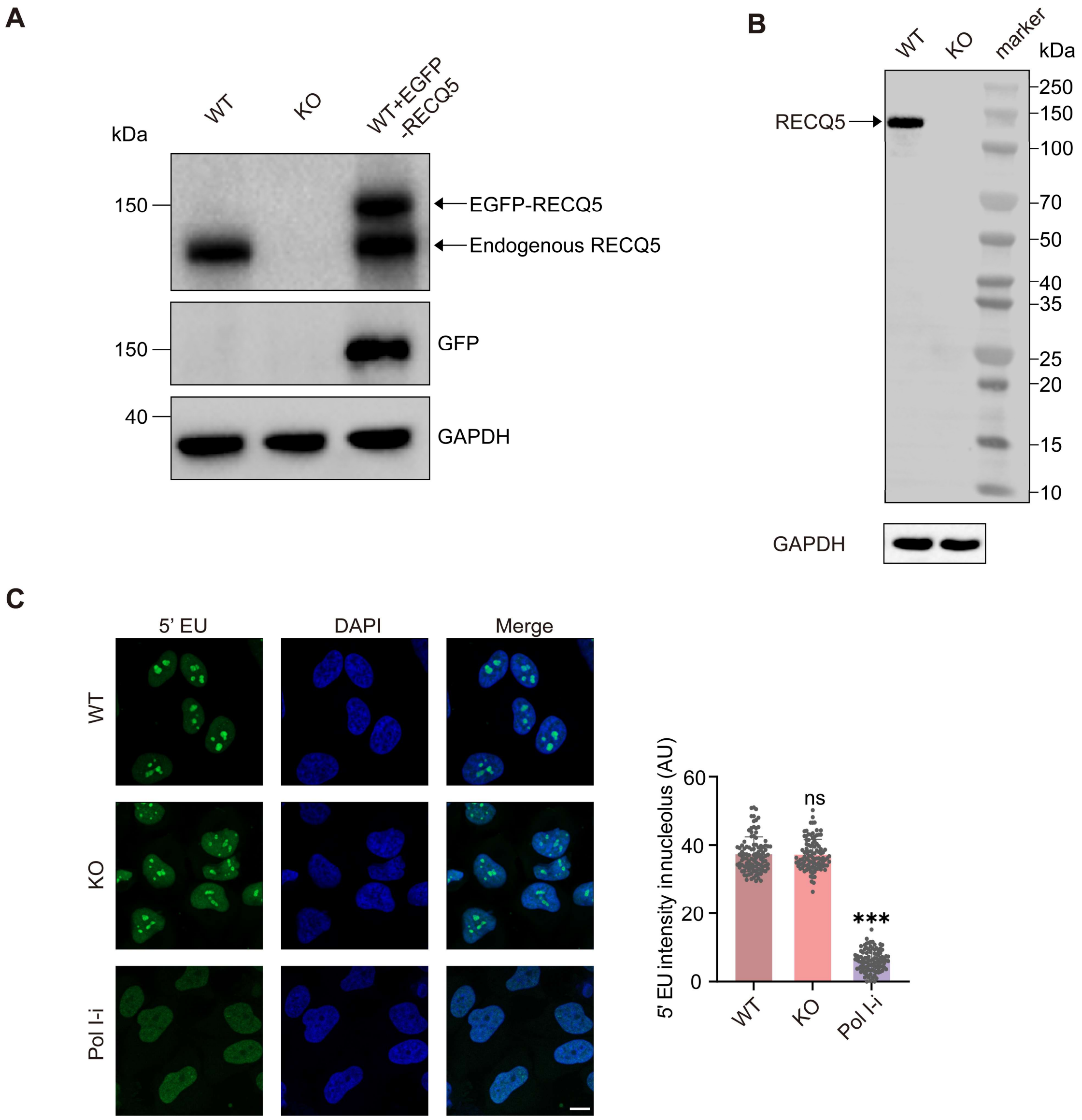

Supplemental Figure S3. RECQ5 depletion does not affect rRNA transcription.

(A) The verification of the anti-RECQ5 antibody. A side-by-side comparison of lysates from RECQ5-WT, RECQ5-KO, and RECQ5-WT expressing EGFP-RECQ5- cells using anti- RECQ5 antibody and anti-GFP antibody. **(B)** RECQ5 is deleted using the CRISPR-Cas9 system in HeLa cells. Western blotting was performed with an anti-RECQ5 antibody. GAPDH was used as a protein loading control. **(C)** Loss of RECQ5 does not affect 5'EU incorporation in the nucleolus. 5'EU was examined by IF. The

fluorescence signal intensity was quantified. n=100 nucleoli per group. Scale bar, 10 μm. RNA Pol I inhibitor (Pol I-i, BMH-21 1 μM) treatment was used as a positive control. Student's t-test is used to determine statistical significance. ns, not significant; ***, $p < 0.001$; ns, not significant.

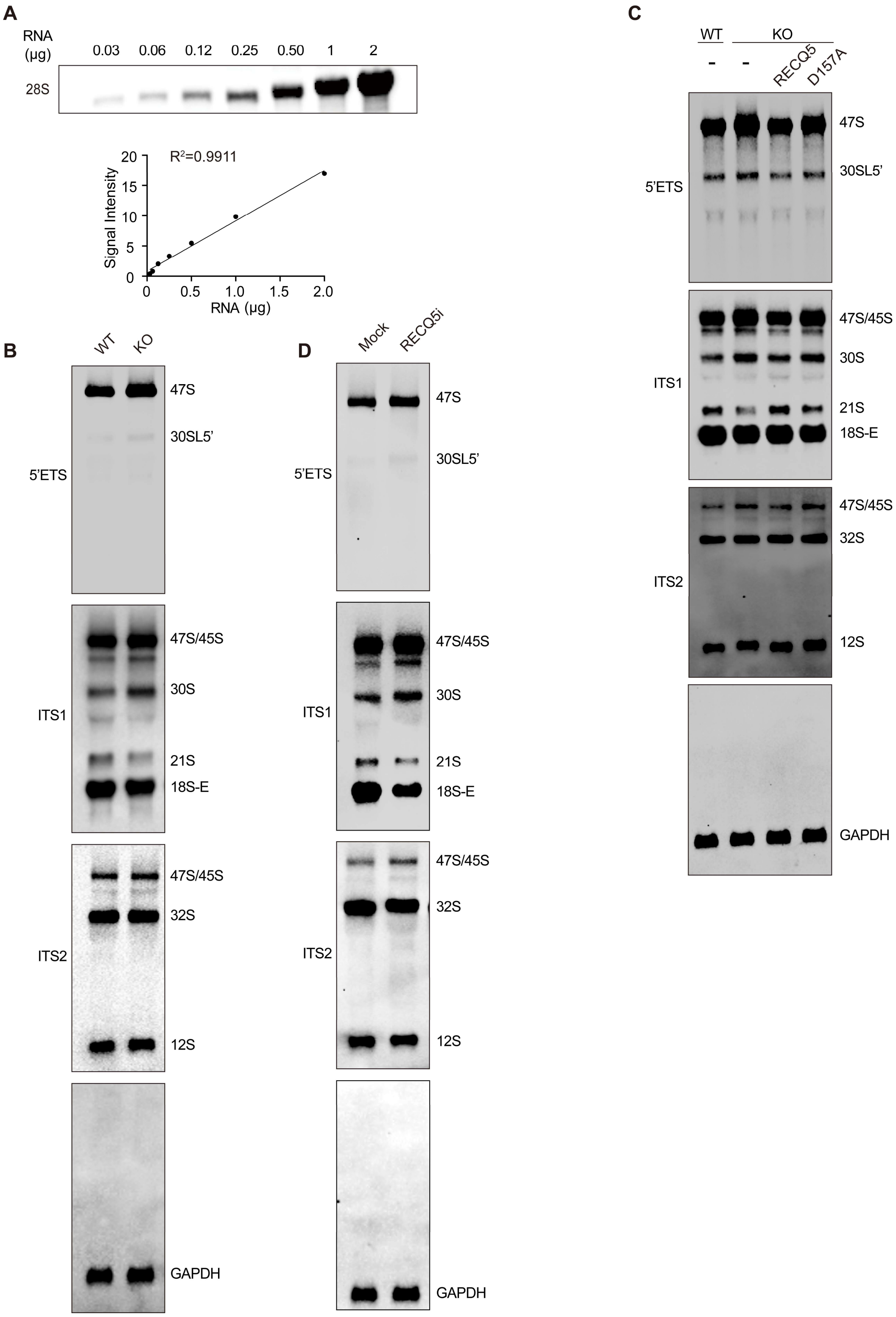

A
RNA (μg)
0.03
0.06
0.12
0.25
0.50
1
2
28S
R²=0.9911
Signal Intensity
RNA (μg)
B
WT
KO
5'ETS
47S
30SL5'
ITS1
47S/45S
30S
21S
18S-E
ITS2
47S/45S
32S
12S
GAPDH
C
WT
KO
-
-
RECQ5
D157A
5'ETS
47S
30SL5'
ITS1
47S/45S
30S
21S
18S-E
ITS2
47S/45S
32S
12S
GAPDH
D
Mock
RECQ5i
5'ETS
47S
30SL5'
ITS1
47S/45S
30S
21S
18S-E
ITS2
47S/45S
32S
12S
GAPDH

Supplemental Figure S4. Loss of RECQ5 impairs pre-rRNA processing in HeLa cells.

(A) A linear correlation between the readout of band density and the amount of loading RNA in Northern blotting. Northern blotting was performed with gradient amounts of RNA, and analyzed with the 28S probe. **(B)** Loss of RECQ5 impairs pre-rRNA processing. Northern blotting was performed to examine pre-rRNA species in HeLa cells. GAPDH was used as the loading control. The blots are uncropped images of Figure 2B**.** **(C)** RECQ5 regulates the processing of pre-rRNA through its helicase activity. Northern blotting was performed in HeLa cells deleted endogenous RECQ5 and reexpressing ectopic RECQ5, helicase-dead mutant D157A. The blots are uncropped images of Figure 2C**.** **(D)** RECQ5 inhibitor treatment impairs pre-rRNA processing. HeLa cells were treated with RECQ5-IN-1 (5 μM) for 24 h. Northern blotting was performed to detect pre-rRNA intermediates. The blots are uncropped images of Figure 2D**.**

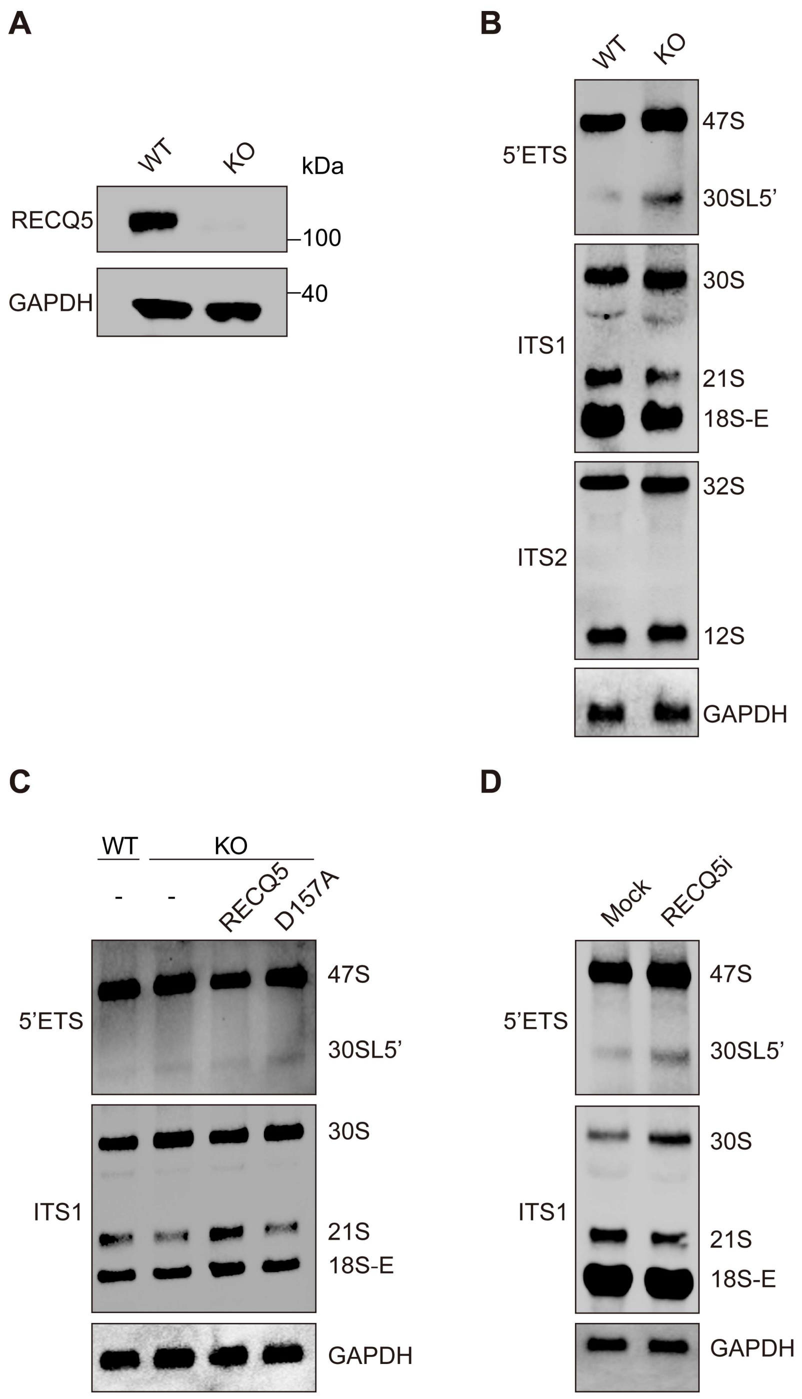

Supplemental Figure S5. Loss of RECQ5 impairs pre-rRNA processing in U2OS cells.

(A) RECQ5 is deleted using the CRISPR-Cas9 system in U2OS cells. GAPDH was used as a protein loading control **(B)** Loss of RECQ5 depletion impairs pre-rRNA

processing. Northern blotting was performed to examine pre-rRNA species in WT and RECQ5-KO U2OS cells. GAPDH was used as the loading control. **(C)** RECQ5 regulates the processing of pre-rRNA through its helicase activity. Northern blotting was performed in U2OS cells deleted endogenous RECQ5 and reexpressing ectopic RECQ5 or helicase-dead mutant D157A. **(D)** RECQ5 inhibitor treatment impairs pre-rRNA processing. U2OS cells were treated with RECQ5-IN-1 (5 μM) for 24 h. Northern blotting was performed to examine pre-rRNA species.

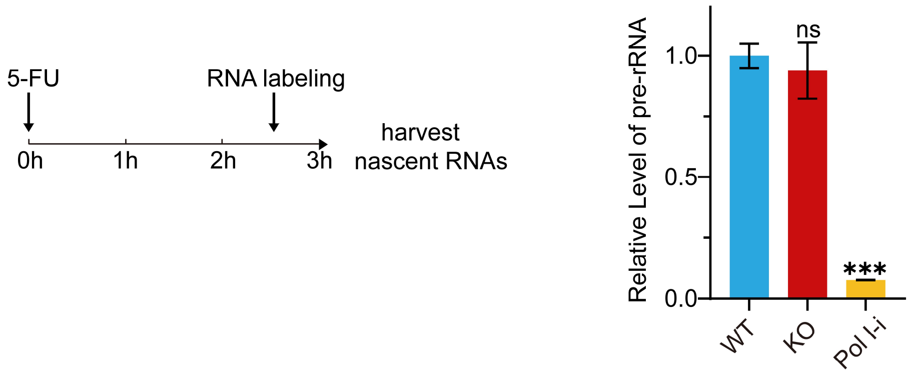

Supplemental Figure S6. Nascent pre-rRNA transcription is not affected in RECQ5-KO cells.

RECQ5-WT and RECQ5-KO cells were pretreated with 5-FU for 3 hours to abolish pre-rRNA processing, and 4sU RNA labeling was performed during the final 0.5 hour. Nascent pre-rRNA was subsequently collected for RT-QPCR and normalized to actin. Pol I-i treatment was used as a positive control.

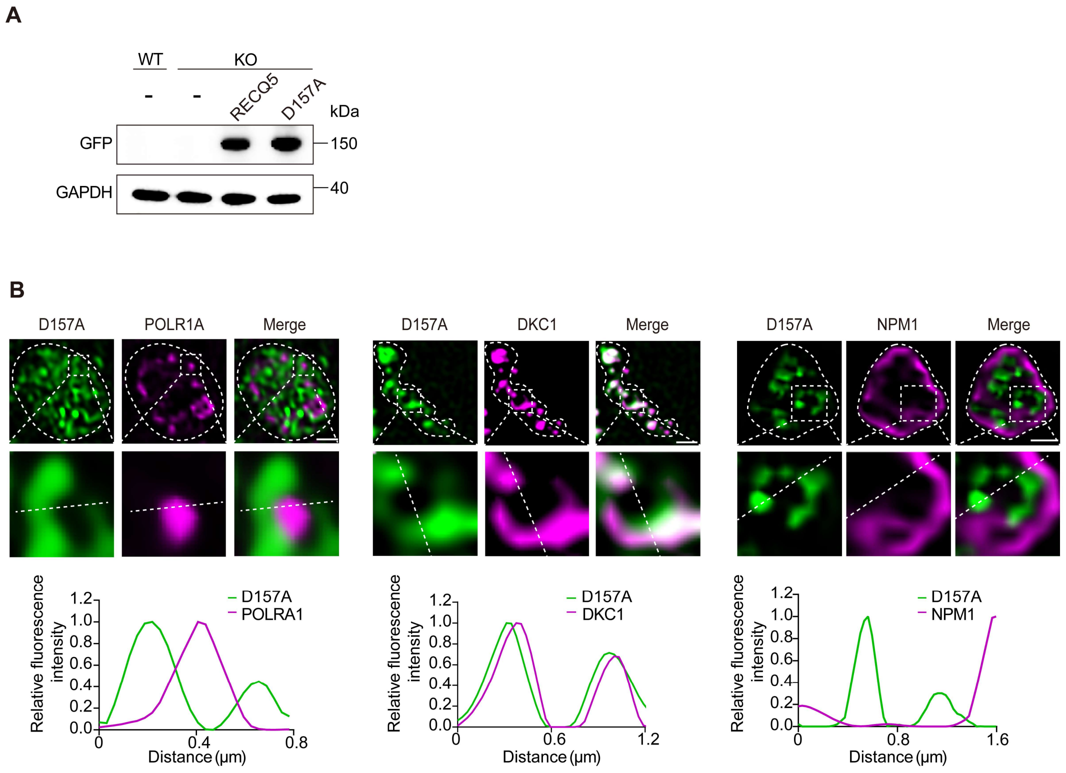

Supplemental Figure S7. The D157A mutation does not affect the expression and nucleolar localization of RECQ5.

(A) Western blots showing expression of GFP-tagged WT-RECQ5 and D157A. **(B)** EGFP-D157A and nucleolar markers of FC, DFC and GC were examined by SIM. The fluorescence intensity on the white dash line was plotted (lower panels). Scale bar, 1 μm.

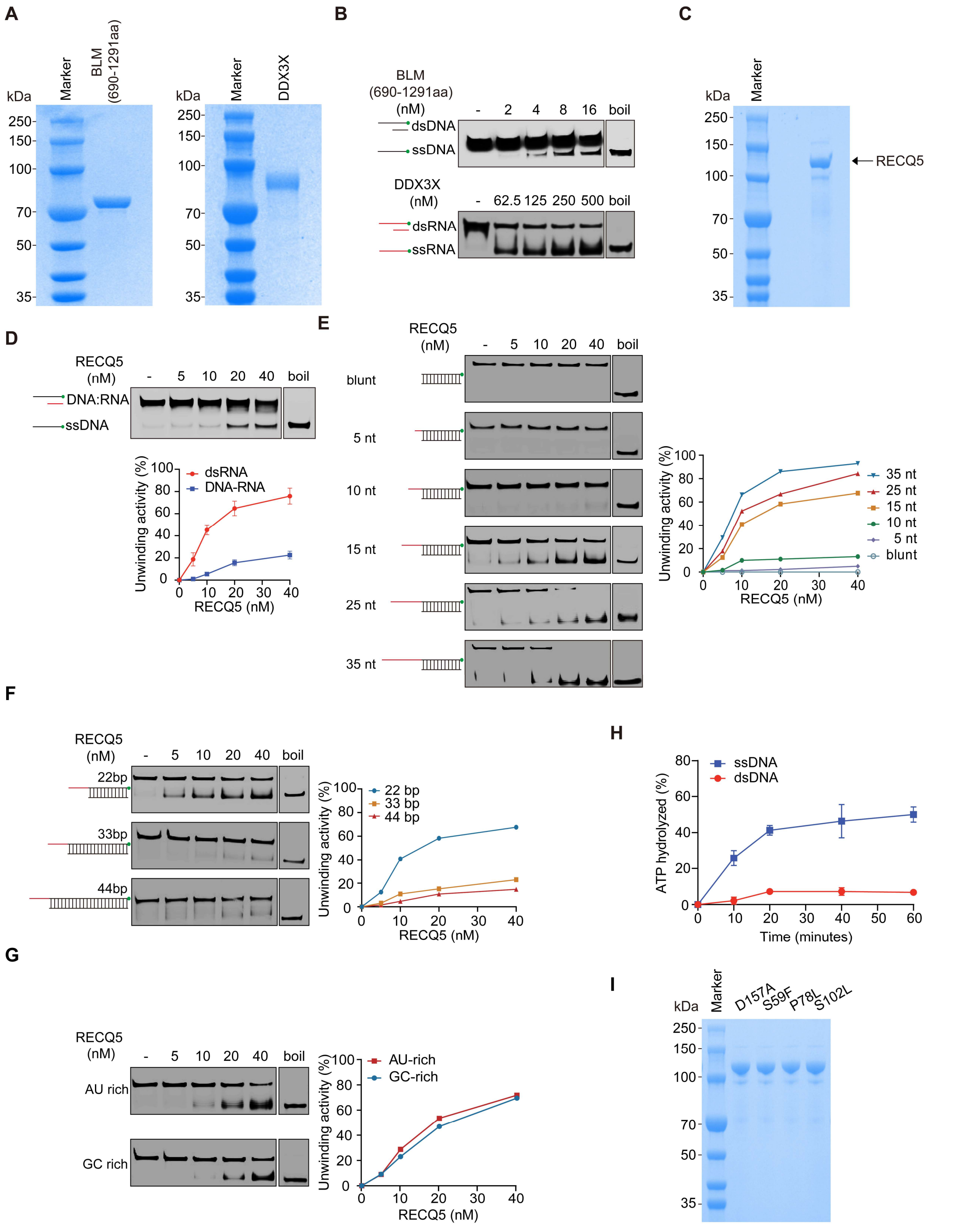

Supplemental Figure S8. The helicase activities of RECQ5. (A) Recombinant BLM (690-1291 aa) is generated from E. coli. The recombinant BLM (690-1291 aa) was examined using gel electrophoresis and stained with Coomassie Blue**.** Recombinant

DDX3X is generated from HEK293. **(B)** BLM (690-1291 aa) unwinds the dsDNA. Recombinant BLM (690-1291 aa) was incubated with dsDNA. DDX3X unwinds the dsRNA. DDX3X was incubated with dsRNA. The reaction products were analyzed using gel electrophoresis. **(C)** Recombinant RECQ5 is generated from E. coli. The recombinant RECQ5 was examined using gel electrophoresis and stained with Coomassie Blue. **(D)** RECQ5 has a weaker unwinding activity on the DNA: RNA hybrid compared with the dsRNA substrate. Recombinant RECQ5 was incubated with DNA: RNA hybrids. The reaction products were analyzed using gel electrophoresis. **(E-G)** Recombinant RECQ5 was incubated with dsRNA substrates with different lengths of 3' overhang (**E**), different lengths of duplexes (**F**), and different GC content (**G**). The reaction products were analyzed using gel electrophoresis. The position of Cy5 label was indicated (green ball). **(H)** RECQ5 does not consume ATP in the presence of dsDNA. Recombinant RECQ5 was incubated with ssDNA or dsDNA. The ATP levels were measured. **(I)** Recombinant RECQ5 mutants are generated from E. coli. The recombinant RECQ5 mutants were examined using gel electrophoresis and stained with Coomassie Blue.

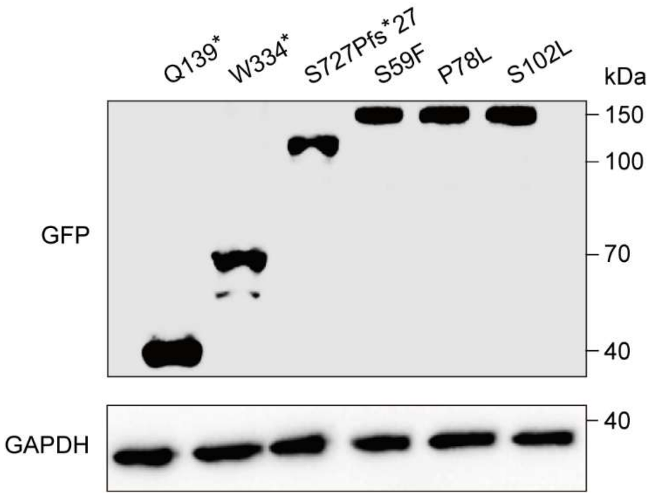

Supplemental Figure S9. Western blots showing expression of GFP-tagged RECQ5 mutants in U2OS cells.

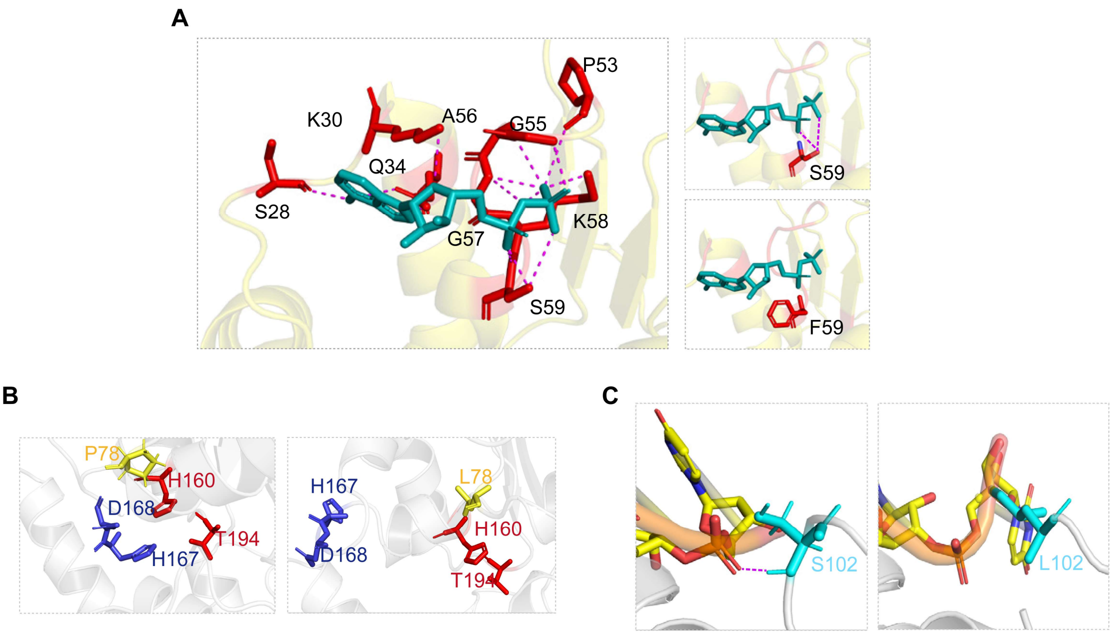

Supplemental Figure 10. Structural analysis of residues involved in ATP hydrolysis and RNA binding.

(A) The S59F mutant disrupts ATP (ADP) binding. Left: The detailed ATP (ADP) binding site in RECQ5 (PDB:5LB3). ATP (ADP) is shown in blue. The ATP (ADP) binding sites are shown in red. The Magenta dash indicated interaction through hydrogen bonds. Right: S59F mutation abolishes the interaction with ATP (ADP). **(B)** The P78L mutant causes conformational changes. Left: the conformations of H160, H167, D168 and T194 are shown. Right: The P78L mutation abolishes the conformation. **(C)** The S102L mutant disrupts the RNA-binding site. Left: S102 directly recognizes RNA. Right: L102 abolishes the RNA-binding. RNA is shown in cyan.

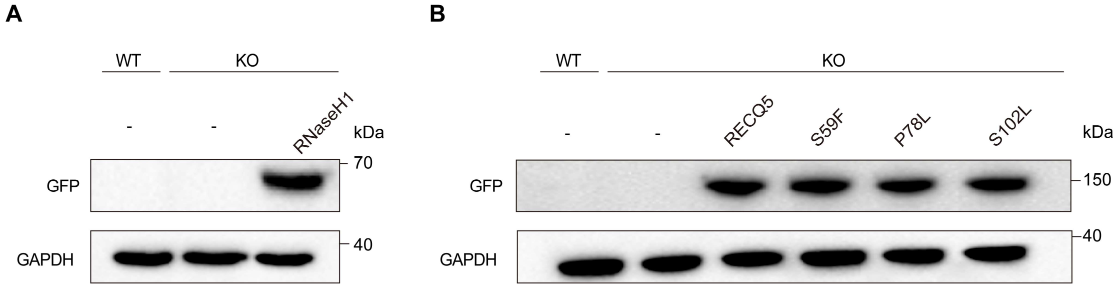

Supplemental Figure S11. Western blots showing expression of GFP-tagged RNaseH1 (A) and the mutants of RECQ5 (B) in RECQ5-KO cells. GAPDH was used as a protein loading control.

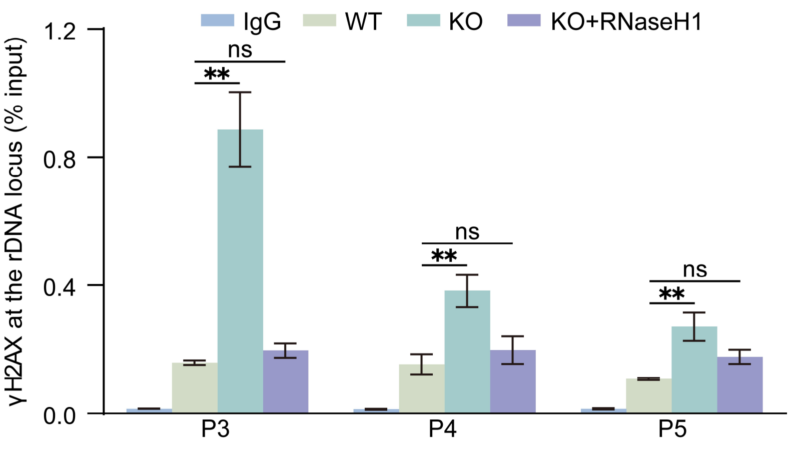

Supplemental Figure 12. Loss of RECQ5 induces the enrichment of γH2AX at rDNA loci.

ChIP-qPCR was performed with γH2AX antibody and indicated primers. The assays were triplicated. Data are represented as means ± SD as indicated. Two-tailed student's t-test is used to determine statistical significance. **, $p < 0.01$; ns, not significant.

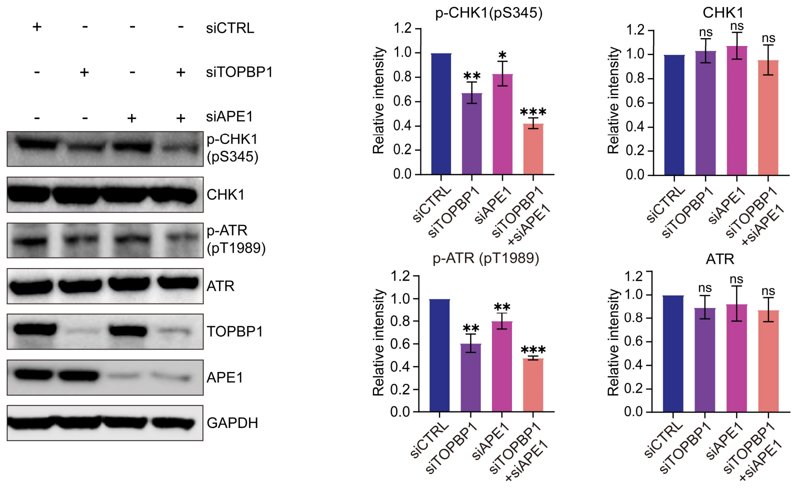

Supplemental Figure S13. RECQ5 depletion-induced ATR Activation is dependent on TOPBP1.

Following knocking down TOPBP1 and APE1 by siRNA in RECQ5-KO cells, Western blotting was performed with the indicated antibodies. GAPDH was used as a protein loading control. The quantitative analysis (relative to GAPDH) was performed. Data are represented as means ± SD as indicated from three independent experiments. Two-tailed Student's t-test was used to determine statistical significance. *, $p < 0.05$; **, $p < 0.01$; ***, $p < 0.001$; ns, not significant.

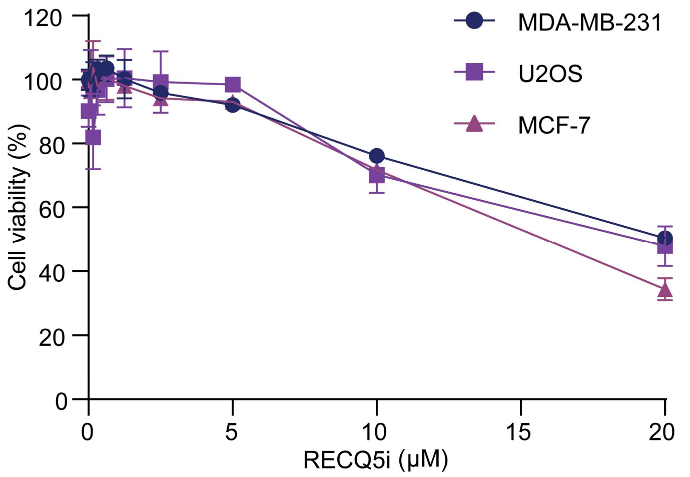

Supplemental Figure S14. RECQ5i treatment on cancer cell lines.

A panel of cancer cells was treated with the indicated dose of RECQ5i for 4 days. Cell viability was examined by CTG assays.
